# Endophytes and Halophytes to Remediate Industrial Wastewater and Saline Soils: Perspectives from Qatar

**DOI:** 10.3390/plants11111497

**Published:** 2022-06-02

**Authors:** Bassam T. Yasseen, Roda F. Al-Thani

**Affiliations:** Department of Biological and Environmental Sciences, College of Arts and Sciences, Qatar University, Doha P.O. Box 2713, Qatar; ralthani@qu.edu.qa

**Keywords:** bacteria, bioremediation, biotechnology, desalination, halophytes, heavy metals, phytoremediation, salt resistance

## Abstract

Many halophytes are considered to be salt hyperaccumulators, adopting ion extrusion and inclusion mechanisms. Such plants, with high aboveground biomass, may play crucial roles in saline habitats, including soil desalination and phytoremediation of polluted soils and waters. These plants cause significant changes in some of the soil’s physical and chemical properties; and have proven efficient in removing heavy metals and metabolizing organic compounds from oil and gas activities. Halophytes in Qatar, such as *Halopeplis perfoliata*, *Salicornia europaea*, *Salsola soda*, and *Tetraena qatarensis*, are shown here to play significant roles in the phytoremediation of polluted soils and waters. Microorganisms associated with these halophytes (such as endophytic bacteria) might boost these plants to remediate saline and polluted soils. A significant number of these bacteria, such as *Bacillus* spp. and *Pseudomonas* spp., are reported here to play important roles in many sectors of life. We explore the mechanisms adopted by the endophytic bacteria to promote and support these halophytes in the desalination of saline soils and phytoremediation of polluted soils. The possible roles played by endophytes in different parts of native plants are given to elucidate the mechanisms of cooperation between these native plants and the associated microorganisms.

## 1. Introduction

In 1980, Epstein et al. [1] stated: “The problem of salinity is an ancient one, but it demands contemporary and innovative approaches”. Thus, the debate about the salinity problem always starts from the depths of history. This problem was first recognized approximately 3000 years BC in Mesopotamia (currently known as Iraq). During the last five decades, many articles have reported how the demise of Sumerian Civilization was attributed at least in part to the salinity problem. Notably, the Sumerian Civilization is not the only one whose history is related to salinity problems, as other examples were reported by many authors [2]. Such historical background has drawn us to discuss the roles of native plants, including halophytes, in removing toxic ions, such as Na^+^, Cl^−^, and heavy metals, as well as metabolizing organic compounds found in saline soils or lands contaminated with industrial wastewaters (IWWs). Such plants were recognized as soil-exhausting plants; they might have developed various structural features, physiological activities, and biochemical pathways associated with their ability to resist saline soils in salt marshes and Sabkhas [3]. These methods and mechanisms include ion compartmentation, production of compatible solutes, salt glands and bladders, and succulence features in the shoot system. Moreover, these plants proved efficient in saline agriculture to provide various useful products, such as fodder, medicine, chemicals, ornamentals, aromatics, food oils, and biofuel [4,5]. Some reports have put forward strategies for developing sustainable biological systems that can be used for the cultivation of halophytic crops in saline lands, as a large number of halophytes can be used as cash crops [6]. During the last two decades, the possibility of using halophytes as a source of traits was discussed to contribute to the development of agriculture by introducing halophytic crops to boost the economy. Such efforts should be accompanied by land management and cultivation of saline soils, bearing in mind that such plants offer genetic pools for gene technology programs [5,7,8]. Such projects need substantial efforts to deal with the recycling of some of these plants whenever necessary to avoid any toxic elements entering the food chain in such environments [9]. Finally, other important roles these plants can play are desalination and phytoremediation of saline and polluted soils. Their roles in desalination have been recognized. The following characteristics of the plants are all key requirements to support their usefulness in desalination: they are salt-resistant, are salt accumulators, have high aboveground biomass, and provide high degrees of economic utility (e.g., fuel, fiber, and oil-seeds) [3,6,10]. 

On the other hand, microorganisms associated with, or adjacent to, these plants might play divergent roles, as plants offer different mini-habitats for them: (1) the rhizosphere (zone of influence of the root system), (2) phyllosphere (aerial plant part), and (3) endosphere (internal transport system). Such associations and interactions may be detrimental or beneficial for either the microorganism or the plant, including neutralism, commensalism, synergism, mutualism, amensalism, competition, or parasitism [11]. This article addresses the role of microorganisms associated with the internal transport system (endosphere) and aerial plant part (phyllosphere). Little attention has been paid to these topics in the Arabian Gulf region in general. and in the State of Qatar in particular, especially the role played by endophytes (microorganisms that occupy the endosphere) in supporting halophytes in the phytoremediation of inorganic and organic components of industrial wastewater and saline soils. This situation needs countries of the Arabian Gulf to contribute generously to international efforts to develop new innovative and contemporary approaches to solve problems facing humanity, from food and health to economy. Information about this topic is scarce; therefore, the methodology of this review aims to present available information about endophytic microorganisms around the world to provide a platform for scientists and researchers in Qatar for further studies in the future.

## 2. Mechanisms of Nature: A Brief Glance

Halophytes, as wild plants, can cope with a wide range of environmental conditions, including salinity, drought, extreme temperatures, and can adopt operating methods and mechanisms, which are regulated by their genetic code. In the Arabian Peninsula, 120 halophytic plant species have been recorded [12], and in Qatar, approximately 26 plant species are recognized as the most common halophytes, constituting approximately 7% of the total number of the flora of Qatar [13]. Halophytes thrive and complete their life cycle successfully in high soil salinity of 16 dSm^−1^ (~200 mmol) or even higher. Halophytes are able to absorb large quantities of salts and regulate them in various plant organs. It can be clearly observed that the aboveground biomass of many of these plants is green and succulent, which makes the plants active and capable of dealing with large quantities of the absorbed salts; herein lies the issue of the different mechanisms by which these plants resist high salinity. Some examples from the flora of Qatar are: *Halopeplis perfoliata* (succulent plant, absorbs large amounts of salt), *Tetraena qatarensis* (high aboveground biomass plant, thrives in polluted lands), and more examples are reported by [3]. More features of all halophytes among the flora of Qatar are discussed in many reports, monographs, and research books [13,14], as these plants have different abilities to absorb and store water, build and accumulate organic and inorganic solutes, and develop structures to regulate these components [15]. These halophytes are also good candidates to remediate polluted soils containing heavy metals and petroleum hydrocarbons [3]. Two primary mechanisms that halophytes in Qatar adopt in dry and saline soils have been reported and discussed in many articles and monographs: (1) avoidance mechanisms and (2) tolerance mechanisms [3,16]. Avoidance mechanisms include three secondary mechanisms: (a) exclusion, (b) extrusion, and (c) inclusion (dilution). Tolerance mechanisms involve: (1) osmotic adjustment to maintain positive water balance between plant tissues and soil environment, and (2) osmoregulation inside the plant cells between vacuoles and cytoplasm. These mechanisms, modifications, and methods help halophytes deal with harsh environments, such as salinity, drought, waterlogging, and pollution with heavy metals and petroleum hydrocarbons, among others.

### 2.1. Avoidance Mechanisms

#### 2.1.1. Exclusion Mechanisms

**Figure 1:**
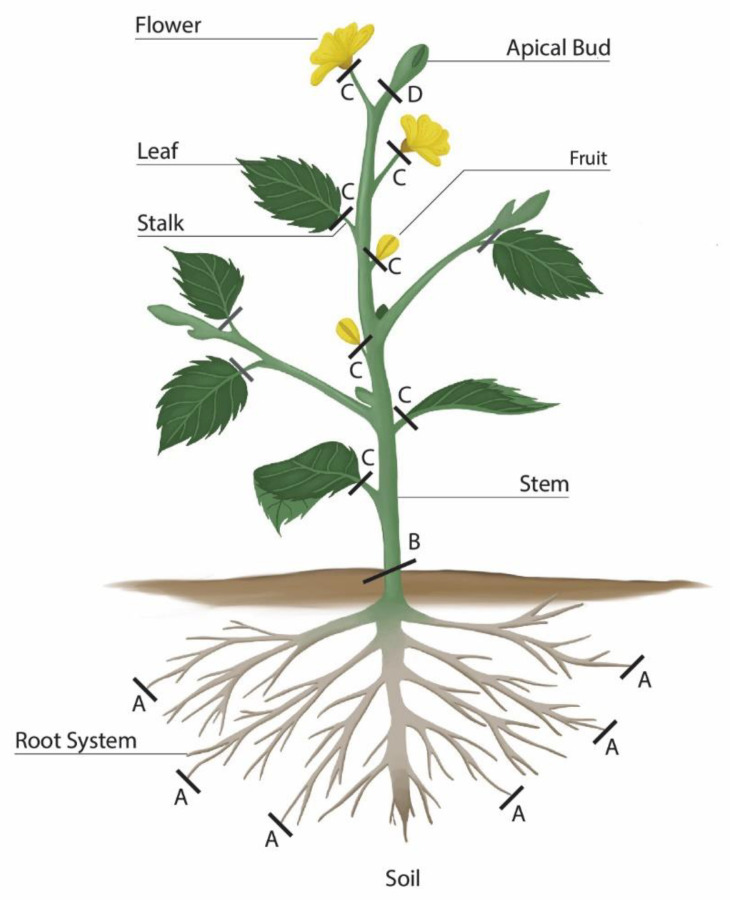
Barriers at different locations of plant organs and tissues as an exclusion mechanism of ions: (A) at the surface of the roots, (B) between shoot system and root system, (C) between leaves and petioles or sheaths, and (D) between apical meristems and the remaining parts of the plant.

Exclusion mechanisms explain how plants have developed presumptive excluding barriers at certain locations along the plant organs to regulate the accumulation of extra ions (e.g., Na^+^, Cl^−^, heavy metals) and to prevent harmful ions of reaching toxic levels inside the sensitive locations of plant tissues. Moreover, exclusion mechanisms may include an intra-ion regulation method to prevent harmful ions from accumulating inside compartments of cells carrying out active metabolic functions; such methods may stand as ion homeostasis inside the plant cells [17,18]. These plants sequester harmful ions to organelles, such as vacuoles, carrying out little metabolic activities. Such methods of ion regulation and osmoregulation inside the plant cells will be discussed with the tolerance mechanism below. Figure 1 shows that interruptions of salt transport take place at particular locations along the plant body, i.e., at the root surface (location A), between stem and root system (location B), between leaves and stalks, between flowers and the stem and branches (location C), and between apical meristems and the remaining parts of the plant (location D), thereby limiting the amounts of salts reaching meristems, developing leaves, and fruits [16]. Such barriers were described in the roots of mangrove plants as filtration systems to prevent the buildup of salts in the conducting system leading to the active green parts of the plant; such merit might attract camels to feed on the green leaves of *Avicennia marina*. Another good example was observed in *Prosopis farcta*; no salt reaches the leaves, although the root system is active in taking up ions such as Na^+^. Such an outcome clearly indicates that some halophytes develop barriers to prevent these ions from reaching high concentrations in leaves and becoming toxic. 

#### 2.1.2. Extrusion Mechanism

**Figure 2:**
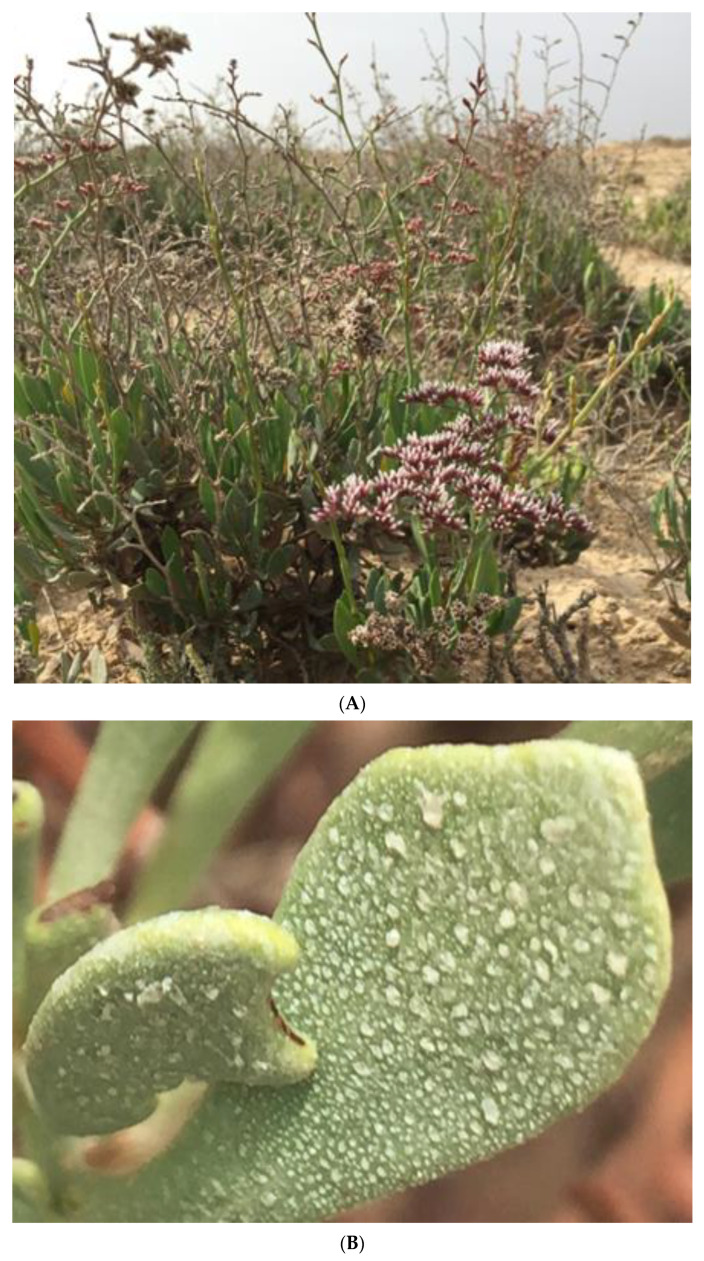
*Limonium axillare* thrives in salt marshes (**A**). Observe the salt crystals on the leaf surface in salt marshes (**B**). Salt glands secrete salts on the leaf surfaces through small holes.

Most halophytes have various structures which are able to eliminate excess salts (Figure 2), and many obligate halophytes, living within Sabkhas and salt marshes, absorb the water they need, accompanied by salt absorption. These plants have structures of three main types: (a) salt glands, (b) salt bladders, and (c) insectivorous salt glands [3,16].

**Figure 3:**
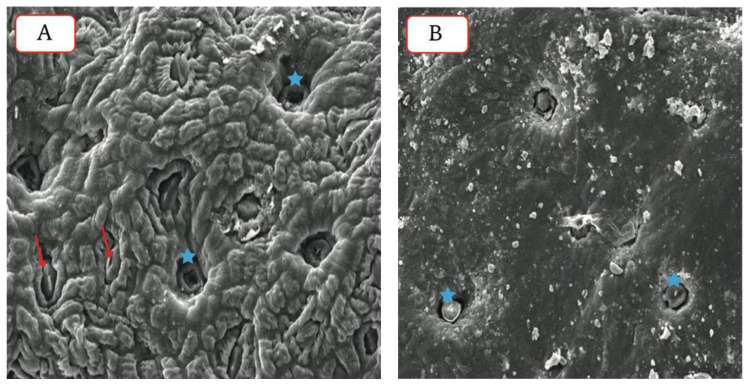

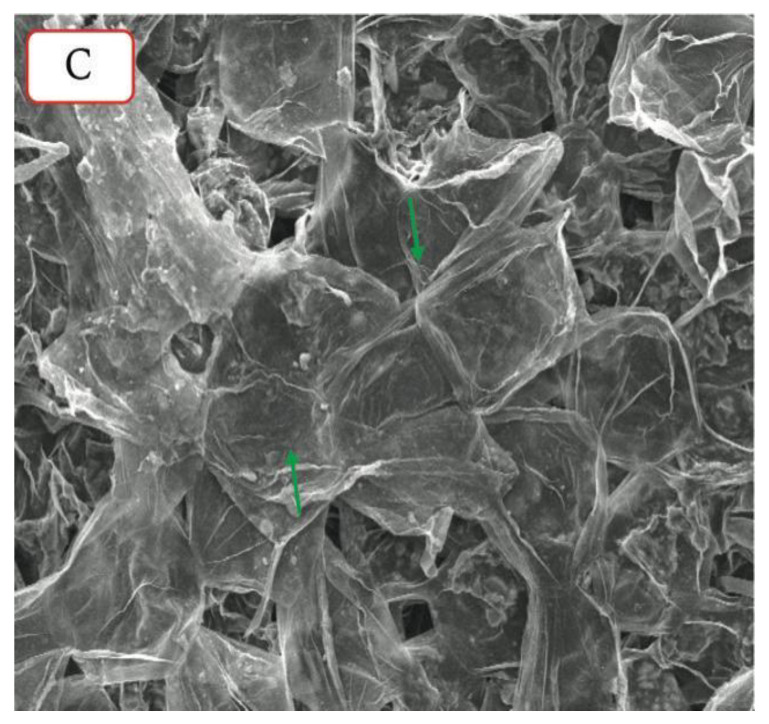
Scanning electron microscope (SEM) images of adaxial (the upper side) leaf surface of (**A**) *Limonium axillare* (note the blue asterisks as salt glands, red arrows as stomata), (**B**) *Avicennia marina* (note the blue asterisks as salt glands, with scattered salt crystals), and (**C**) *Atriplex* spp. (note the green arrows as ruptured salt bladders). Magnification ×400. N.B. Salt glands in *A. marina* are found on both leaf sides but are more numerous abaxially (lower side), small in number and large in size on the adaxial surface, and the opposite on the abaxial surface.

Salt glands are embedded in the leaf surface, and their size approximates that of stomata (Figure 3), reaching as much as 1000 per cm^−2^ on the leaf surface. They differ in the number of cells comprising them. Good examples of salt glands can be found in the genera *Avicennia*, *Frankenia*, *Limonium*, and *Tamarix*, while salt bladders are best represented in *Atriplex* leaf surfaces (Figure S1). The high-water absorption needed by these plants is accompanied by salt absorption; such plants are designed to extrude extra salts through salt glands, salt bladders, and possibly other structures. Moreover, these plants have fleshy leaves, as in *Limonium* and *Atriplex*, to extrude extra salts. 

#### 2.1.3. Inclusion Mechanism

The inclusion mechanism can also be indicated as a dilution mechanism. Succulence is a very common phenomenon in halophytes, but some observations were noticed in glycophytes as well [19]. These succulent plants absorb significant amounts of toxic ions (Na^+^, Cl^−^, and possibly others) as an inclusion mechanism aiming to remove substantial amounts of salts from saline soils. Succulence as an avoidance mechanism takes place when the extra ions, such as Na^+^ and Cl^−^, are not excluded, re-translocated, or extruded. Instead, in the avoidance mechanism of succulence, extra ions are sufficiently diluted in the shoot system, especially the leaves, to keep the cytoplasmic salinity below toxic levels, and the ions are sequestered in the vacuoles of mesophyll tissues. Plants such as *Anabasis*, *Arthrocnemum*, *Atriplex*, *Halocnemum*, *Halopeplis*, *Limonium axillare*, *Salsola*, *Suaeda*, and *Tetraena qatarensis*, among other plants, are good examples from the Qatari flora (13, 14); these are halotolerant inclusion mechanism-adopting plants because they absorb significant amounts of Na^+^ and Cl^−^ ions, establishing the phenomenon of succulency [14,19].

In fact, high internal NaCl levels are compensated by high water storage, leading to a high proportion of water to dry weight. Therefore, it is believed that as soil salinity rises, the succulence of these plants increases as both water and salt absorption increase [20]. Therefore, most halophytes exhibit one or more of the avoidance mechanisms (exclusion, extrusion, and dilution). The last two mechanisms are adopted to cope with the potential ability of halophytes to absorb substantial amounts of salts from the environment. However, there is no evidence yet that halophytes, having a clear succulence phenomenon, have other avoidance mechanisms to cope with high soil salinity. 

### 2.2. Tolerance Mechanism

The tolerance mechanisms are developed in many halophytes to deal with one major issue, i.e., the absorption of large quantities of salts, as an inevitable consequence of their adaptation to saline soils. Na^+^ and Cl^−^, the most abundant ions in the soil environment of halophytes, are accumulated inside the plant tissues to achieve osmotic adjustment with the plant environment [21,22]. Other physiological and biochemical activities can be carried out to lower the water and solute potentials of plant cells by accumulating organic and inorganic solutes. Moreover, osmoregulation is another activity conducted by plant tissues to maintain ion homeostasis inside plant cells and to regulate inorganic ions, including the toxic ones inside plant cells, through sequestration of Na^+^, Cl^−^, and possibly others, in the vacuoles, and the biosynthesis and accumulation of organic components, such as compatible solutes, proline, glycinebetaine, sugars (e.g., trehalose), and polyols at the cytoplasm [23,24,25,26]. The role of these compatible solutes to maintain the life of these plants in their natural habitats is well documented [26,27,28,29,30]. However, it is not the objective of this review article to discuss the functional details of these organic solutes in these plants. Regardless, these plants are able to remediate soils and water and remove toxic ions and pollutants from marshes and saline soils [9,31].

## 3. Phytoremediation in Saline and Polluted Soils

Halophytes in Qatar are found mainly at the coastlines and Sabkhas. Others thrive in isolated areas created after heavy rains on saline and dry soil. Notably, most of these halophytes are perennial succulents, semi-woody dwarf shrubs, belonging to the families of Amaranthaceae, Cyperaceae, Juncaceae, Plumbaginaceae, Poaceae, Zygophyllaceae, and others [13]. Interestingly, these halophytes grow and thrive on land with active oil and gas activities. Such natural concurrence between industrial activities and the presence of such native plants inspires scientists, and research centers, to examine the roles of these plants in the polluted soils. Recent studies have discussed the role of many native plants in the Arabian Gulf region in general, and in Qatar in particular. These studies included the following topics: (a) solute accumulation in response to pollution with organic and inorganic components due to oil and gas activities [30], (b) phytoremediation of polluted soils and waters from heavy metals and petroleum hydrocarbons [9], and (c) bioremediation and phytoremediation roles of microorganisms at rhizosphere and phyllosphere, as a biological approach to remediate soils and purify water; providing an alternative source of future water in this region [31]. During the last two decades, however, some evidence has been presented that adjacent or associated microorganisms coexisting with these halophytes might support their roles in the phytoremediation of contaminated and saline soils. To elucidate the role of halophytes in polluted habitats, the following topics will be discussed below: (A) desalination of soil, (B) detoxification of polluted soils. Regarding detoxification of polluted soil the following is addressed (1) bio-mining of polluted soils and (2) metabolizing of petroleum hydrocarbons, (C) roles of adjacent and associated microorganisms, horizontal gene transfer (HGT), and (D) modern biotechnology, which includes the genetic approach.

### 3.1. Desalination of Soil

One of the primary strategies for increasing crop production and improving agriculture under a saline environment is environmental manipulation. By improving the soil conditions, the strategy of “better soil for crops we have” is implemented without manipulating the genetics of the crops we have. Indeed, such a strategy was suggested as a possible way to achieve that goal; it is based on the implementation of a large scheme of (a) irrigation with high-quality water, (b) conservation of existing agricultural lands, (c) reclamation methods, such as constructing good drainage systems, and (d) application of supplementary irrigation in lands having uncertain and unguaranteed rainfall [32]. However, almost all these measures might not be applicable to Qatar and other countries in the region and are not easy approaches in terms of money, energy, labor, and sustainable success for the long run [1,30]. Unfortunately, these methods can not only eliminate harmful ions from a saline environment but may also remove essential elements. Therefore, soil conditions after these measures need substantial care and the application of special agricultural practices. Furthermore, such soils need large-scale applications of fertilizers. In the end, salts can accumulate by continuous irrigation, causing a salinity problem again. Moreover, mechanical seawater desalinization processes to support the agricultural sector and provide drinking water are expensive [9], and it is not feasible to use such water for reclamation processes. Storing good quality water in strategic reservoirs has been conducted to achieve one important goal: to support the people’s needs during emergencies and crises when the country is hit by future unseen threats [9,31]. Therefore, irrigation of crops and expanding the cultivation of agricultural lands in these regions might lead to the use of low-salinity water, and such water could include the use of treated sewage water and brackish water. In practice, low salinity water could be used to supplement high-quality irrigation water. This would permit the expansion of irrigated agriculture and provide a means of partially disposing of saline drainage water and anthropogenic wastewater. However, the risk of accumulating salt in those lands is still very high. Therefore, environmental approaches do not offer real solutions to the problems facing agriculture in the Arabian Gulf region at the present time.

Halophytic plants are good and promising candidates to clean the environment from most kinds of pollution. Studies have been carried out, and many articles have been published, to show a new era of using halophytes for the phytoremediation of saline soils as a new approach to solving the problems facing agriculture and wildlife. Many native plants proved efficient in remediating polluted soils and waters containing heavy metals and petroleum hydrocarbons; such approaches are environmentally friendly for many problems facing the ecosystem and human life in health, agriculture, and economy [9,31]. Early reports [32] recommended the selection of appropriate native plants to restore such soils. Thus, the following discussion is dedicated to the possibility of cultivating such plants, including halophytes, in saline soils to remove toxic ions, such as Na^+^ and Cl^−^, leading to successful reclamation of polluted saline lands. Desalination of soils and waters has become an inevitable option to remove toxic salts, including heavy metals, as it has become a preoccupation for the expansion and revitalization of the agricultural sector. Scientists have indicated many reasons behind such efforts:

Soil salinity and pollution have become a source of serious concern facing the agricultural economy, not only in this region, but worldwide as well [31].

Irrigation with quality water has become a problem in many countries worldwide [33], and this is even worse in the Arabian Gulf States.

It is necessary to improve the physicochemical properties of the soil, to provide good conditions for crop plants and microbes to work together to boost the cultivation of lands and to increase efficiency of phytoremediation [34,35].

Halophytes, which can survive and reproduce in high-salt environments, accumulating and extruding large amounts of Na^+^ and Cl^−^ ions, could be used as food crops through saline water irrigation, and are potentially ideal candidates for phytoremediation of heavy metal-contaminated saline soils as well [36,37]. Such an ironic and tricky point was addressed in many articles through active monitoring systems involving such plants in recycling and industrial activities [9]. Indeed, Al-Thani and Yasseen [9] gave more details about such an issue. Phytoremediation actions by plants are classified into three groups: (a) not preferred and not recommended for edible plants (crops and fruits), (b) preferred after monitoring, this group included native plants not edible for humans but considered as fodder for livestock, and (c) preferred for native plants not edible by neither humans nor livestock. The groups b and c contain native plants including halophytes.

Lack of arable land, due to salinity and pollution, makes it the duty of scientists to adopt modern methods and techniques, and for decision-makers to take the initiative and implement all the necessary measures and legislations to take serious steps with the main goal of getting benefit from the land after ridding it of salinity and pollution. Such land can then be cultivated with major crops [38]. Biological approaches and biotechnological methods are promising strategies to achieve these objectives [3]. 

Looking at the native halophytes among the flora of Qatar, many plants proved efficient in desalination and reclamation of salt-affected lands. For example, Ajmal Khan and Gul [36] showed that *Arthrocnemum meridionale* has a high degree of salt tolerance and could accumulate large quantities of Na^+^ and Cl^−^ ions. Hasanuzzaman et al. [39] compared environmental manipulation, in terms of agronomic practices, with the biological approach using halophytes to remediate saline soils and remove harmful ions. However, successful environmental manipulation using agronomic practices is costly and needs intensive labor and a comprehensive system of monitoring and follow-up. Moreover, salt-tolerant glycophytes (some crops, such as date-palm trees, sugar beet, barley, etc.) do not fully meet the requirements of successful phytoremediation, as most of these plants lack specialized anatomical features to extrude salts, with limited inclusion mechanisms. Instead, these plants have developed exclusion mechanisms with varying effectiveness at certain locations through the plant body, as shown in Figure 1 [40]. Most of these plants have limited exclusion mechanisms to prevent salts entering the shoot system or to exclude harmful ions to the root environment (a mechanism operating largely in *Phoenix dactylifera* (date-palm trees) [41] or accumulating salts in plant organs carrying little metabolic activities, such as leaf petioles, stalks, and sheaths [16,42] (Figures S2 and S3). Thus, Hasanuzzaman et al. [39] listed many halophytes, including grasses, shrubs, and trees, that have various resistance mechanisms (avoidance and tolerance) to remove salts from different polluted saline soils. These plants and others listed in [43] as efficient native halophytes in the UAE to re-acclimate salt-affected lands include: *Arthrocnemum meridionale*, *Atriplex* spp., *Avicennia marina*, *Halocnemum strobilaceum*, *Halopeplis perfoliata*, *Haloxylon* spp., *Salicornia* spp., *Salsola* spp., *Sporobolus virginicus*, and *Suaeda* spp. *Tamarix aphylla*, *Zygophyllum* spp. Many of these plants have salt glands or salt bladders, adopting extrusion methods of the avoidance mechanism. Halophytes having salt glands are well represented in the Qatari ecosystem by *Tamarix*, *Limonium*, and *Frankenia*. The amount of the excreted salts was estimated in some of these plants; each gland or bladder may excrete up to 0.5 μL of salt solution in an hour [44]. Such findings should encourage researchers to conduct comprehensive studies to estimate the salts excreted from these structures. The outcomes of such studies should be generalized and utilized for future phytoremediation projects to clean up high salt contaminated soils. Table 1 shows more halophytes in the Qatari ecosystem that are able to remediate saline-polluted soils. Many of them have either succulent leaves or stems, and in some other cases, the whole plant is succulent, which means many of these plants are able to accumulate multiple salts by adopting a dilution mechanism [14]. As far as the phytoremediation of saline lands is concerned, many halophytes in Qatar are good candidates for cleaning the salty soils of toxic ions, such as Na^+^ and Cl^−^, still, further studies are needed to look at the potential of other halophytic plants. Moreover, these native plants proved to play other roles in the Qatari ecosystem that need to be explored. The following roles and activities have been reported:

As food crops, many halophytes in the Qatari lands are edible for livestock and cattle as forage of good value [45].

As medicinal plants, some of the halophytes listed in Table 1. 

**Table 1:** Halophyte plants among the flora of Qatar and their ability to absorb and accumulate Na^+^ and Cl^−^ ions.

Plants	Habitat & Distribution	Remarks & Roles		References
		Remarks	Roles	
*Aerluropus* spp. (Monocot)	Highly saline sandy soil, shallow Sabkhas	Not succulent, extrusion mechanism with high selectivity to Na^+^	Efficient Na^+^ accumulator, recommended remediator	[46,47]
*Anabasis setifera* (Dicot)	Periphery of Sabkhas, stressed in dry and saline soils	Succulent leaves, it is a facultative halophyte, inclusion mechanism	Accumulates substantial amount of Na^+^ & Cl^−^	[20,48,49]
*Arthrocnemum meridionale* * (Dicot)	Tidal zone and Sabkha depressions	Succulent shoots, inclusion mechanism	Efficient Na^+^ & Cl^−^ accumulator	[36,50]
*Atriplex leucoclada* (Dicot)	Saline sandy soil, Sabkhas, and coastlines	Not succulent, extrusion mechanism	Reduces soil salts (desalination), efficient Na^+^ & Cl^−^ absorption	[51]
*Avicennia marina* (Dicot)	Muddy tidal zone	Not succulent, much accumulation of Na^+^ and Cl^−^, sugar accumulation	Restoration program & desalination	[52]
*Cleome* spp. (Dicot)	Sandy coastal soil	Not succulent	Needs to be evaluated	[53]
*Cressa cretica* (Dicot)	Moist saline soils & Sabkhas	Not succulent, high salt tolerance	Herbal medicine (antibacterial and anti-fungi), possible role of associated bacteria	[54]
*Cyperus* spp. (Monocot)	Coastal saline areas, Agric. fields	Not succulent, tolerance mechanism is operating, medicinal plants	Possible desalination role, revegetation of salt affected lands	[55]
*Frankenia pulverulenta* (Dicot)	Moist saline soils	Not succulent, medicinal plant	Accumulates Na^+^ & Cl^−^, less K^+^	[56]
*Halocnemum strobilaceum* (Dicot)	Salt flats	Succulent shoots	Accumulates Na^+^ & Cl^−^, and remediates saline soil	[14,57]
*Halodule uninervis* (Monocot)	Marine, shallow depths	Not succulent, accumulates Na^+^, Cl^−^, and K^+^	Remediates sea water	[33,58]
*Halopeplis perfoliata* (Dicot)	Highly saline Sabkhas with sandy shelly soil	Succulent shoots, high Na^+^ and Cl^−^ content, accumulation of compatible solutes	Remediate saline patches	[43,59]
*Halopyrum mucronatum* (Monocot)	Coastal dunes	Not succulent, seawater inhibits its germination	Possible remediation role at vegetative stage and bioenergy crops	[60]
*Haloxylon* sp. (Dicot)	Highly saline patches	Succulent stems, highly salt-tolerant, some species are xerophytes	Accumulates Na^+^ & Cl^−^, phytoremediation role is possible	[61]
*Heliotropium* spp. (Dicot)	Saline sandy soil, fields and gardens	Not succulent, found at saline, alkaline, and dry soils	Phytoremediation role is possible	[14,62]
*Juncus rigidus* (Monocot)	Swamp brackish waters	Not succulent	Phytoremediation of organic compounds, heavy metals, and saline soil	[63]
*Limonium axillare* (Dicot)	Coastline with saline shelly soil	Succulent leaves, extrusion mechanism is operating, succulent plant	Useful in Phytoremediation of saline soil	[22,64]
*Polypogon monspeliensis* (Monocot)	Gardens and fields, near the sea shores and salt marshes	Not succulent, suitable for saline soils and rich of Zn	Salinity can alleviate the toxicity of Zn	[65]
*Salicornia europaea* (Dicot)	Muddy salty tidal zones	Succulent, model for salt tolerance studies	Possible saline crop, phytoremediation of salts at constructed wetlands	[66,67]
*Salsola* sp. (Dicot)	Moist saline soil, coastal sand dunes	Succulent, inclusion mechanism is operating, high content of Na^+^ and Cl^−^	Possible phytoremediation of saline soils	[62] (This article covered many halophytes)
*Seidlitzia rosmarinus* (Dicot)	Very well adapted at dry and saline lands	Succulent shoots, inclusion mechanism is operating, high content of Na^+^ and Cl^−^	Phytoremediation of saline soils	[59,68]
*Sporobolus* spp. (Monocot)	Moist saline sandy soils	Succulent, efficient extrusion & inclusion mechanisms are operating	Accumulate compatible solutes at cytoplasm, accumulate Na^+^ & Cl^−^, high root content of K^+^	[69,70,71]
*Suaeda* spp. (Dicot)	Moist saline soil in Sabkhas	Succulent, inclusion mechanism is operating, high content of Na^+^ and Cl^−^	Possible phytoremediation of saline soils	[72]
*Tamarix* spp. (Dicot)	Moist saline soils, fields and depressions	Not succulent, extrusion mechanism is operating, high accumulation of salts	Phytoremediator of saline soils	[73,74]
*Tetraena qatarensis* (Dicot)	Found at many locations of Qatar, coastline, disturbed rocky and sandy areas	Succulent, inclusion mechanism is operating, high content of Na^+^ and Cl^−^	phytoremediator of saline soils	[14,48]
*Teucrium polium* (Dicot)	Saline and shallow depressions	Not succulent, needs confirmation about its phytoremediation activities	Medicinal plant, antimicrobial effects against some microbes	[13,75]

* *Arthrocnemum meridionale* (Ramírez, et al.) Fuente, et al. (previously known as *Arthrocnemum macrostachyum*).

Plants such as Halocnemum strobilaceum, have medical roles to cure many ills [13,45,54,57,75].

As bioenergy crops, the biomass and yield of some halophytes can be utilized as biofuel, for example, *Halopyrum mucronatum* are good candidates as a bioenergy crop. Oil produced from its seeds and the lignocellulosic biomass of this plant can be utilized for biofuel production [60].

As biochemical components, halophytes produced many compatible solutes, such as proline, glycinebetaine, and K [30].

In terms of economic values, some halophytes have high nutritional values as sources of edible oils and production of chemicals [68].

For their ecological roles (perhaps other crucial roles need to be discussed as well), halophytes and their associated microorganisms (bacteria and fungi) might remediate land polluted with heavy metals and organic components [37,76]. Future studies should concentrate on these native plants to examine the possibility of constructing engineered terrestrial land (ETL) to improve the soil conditions for cultivating various crop plants.

### 3.2. Selective Absorption of Toxic Ions

Some lessons can be learned from salt-resistant glycophytes. Sugar beet is a salt-tolerant crop cultivated in many countries worldwide for sugar production. Sodium chloride fertilizers can be used to improve growth, water status, and yield. Early reports showed that the accumulation of chloride in sugar beet leaves was accompanied by an increased cell volume and relative water content (RWC) [19,77]. Although K^+^ is a major nutrient element, it is not found in any synthesized compound of plants and is not replaceable in many cytoplasmic functions. However, early reports showed that some roles of K^+^ might be substituted by Na^+^ or Mg^+2^ accumulated in this plant for some physiological and biochemical functions in the plant; otherwise, some organic solutes might play the roles of Na^+^ or Mg^+2^ in their absence [78,79]. In their early reports, Flowers and Lauchli [78] discussed the possible substitutional roles of Na^+^ for K^+^ in plant cells. They reported the following possible roles: 

Na^+^ may partially alleviate the requirement of the stomatal movement for K.

Na^+^ may contribute to the solute potential and osmoregulation inside the cells and consequently in the generation of turgor. 

Na^+^ is almost as effective as K^+^ for leaf expansion.

Na^+^ may replace K^+^ as an enzyme activator in some metabolic activities. Both Na^+^ and K^+^ are equally effective on malate dehydrogenase activity in maize and barley [80]. 

In barley cultivars, Abu-Al-Basal and Yasseen [81] suggested two possible mechanisms to maintain optimal cytosolic K^+^/Na^+^ ratio in the shoot tissues, and this can be achieved by either (1) restricting Na^+^ accumulation in plant tissues or (2) preventing K^+^ loss from the cell [82,83]. Moreover, early reports have shown active exchange of K^+^-Na^+^ across the young tissues of some plants, such as barley [84]; low concentrations of K^+^ salts around the root tissues induce rapid extrusion of major parts of Na^+^ exchange for K^+^ [85]. The unusual accumulation of K^+^ in leaves of some crops under salt stress was explained by the activation of some transporters, such as high-affinity potassium (K^+^) uptake transporters (HKTs) to maintain high K^+^ levels in the plant tissues [86]. Some other reports have concluded that using low concentrations of NaCl (as a fertilizer) promote the growth of sugar beet plants [77]. Similar reports have shown that low NaCl concentrations (approximately 50 mM) in the growth medium enhance the growth of halophytes (*Atriplex gmelina*), while high levels of KCl salt might have a deleterious effect on growth, as compared to NaCl salt [87]. They concluded that some complex systems operating in these plants could have a great influence on the accumulation of these ions in halophyte plants under saline environments. All these findings and conclusions have drawn attention to opening a forum of discussion about the selectivity some halophytes have and what biotechnology might achieve to develop plants having selective traits for a particular heavy metal at specific polluted lands. However, this objective is still being investigated to reach a final conclusion [88], Personal communication: Flowers, T. J., November 2020. 

## 4. Detoxification of Polluted Soils

Studies during the last decade have warned that anthropogenic and industrial activities and agricultural practices might have left pollutants in the soil [89], especially those resulting from various sectors of industry and expansions in oil and gas investments. Regarding oil and gas, large quantities of accumulated heavy metals and organic compounds, such as petroleum hydrocarbons, surely have a negative impact on various sectors of agriculture, health, and wildlife. Such issues, which could affect the coastline and underground water, should sound an alarm in a small country like Qatar. Polluted water at these locations might affect various life sectors, especially those related to agriculture and domestic purposes. Native plants, including halophytes, that can resist highly saline soils while completing their life cycles and reproducing in such a harsh environment, are potentially ideal for phytoremediation of soils contaminated with heavy metals and organic components [37]. Indeed, a biological approach using such plants might be useful to remediate soil and water not only from salts (Table 1), but also from various pollutants, such as heavy metals and petroleum hydrocarbons. Thus, detoxification of these components is a prerequisite for successful ecological restoration and maintenance of a healthy environment [31]. 

### 4.1. Bio-Mining of Polluted Soils

Industrial wastewater (IWW) from oil and gas activities at the Arabian Peninsula in general, and in Qatar in particular, contain a large number of heavy metals, such as Al, As, Ba, Cd, Co, Cr, Cu, Fe, Hg, Mn, Ni, Mo, Pb, V, Zn, and possibly others that are present at low concentrations [31,90,91,92]. The study of Al-Khateeb and Leilah [93] listed many halophytes that efficiently accumulate most of these heavy metals. These plants included *Anabasis setifera*, *Cyperus* spp., *Halocnemum strobilaceum*, *Haloxylon* sp., *Panicum turgidum*, *Pennisetum divisum*, *Salsola* spp., *Seidlitzia rosmarinus*, *Suaeda* spp., and *Zygophyllum* spp. Therefore, phytoremediation processes are necessary for successful ecological restoration and maintenance of a healthy environment. Native plants, including halophytes, could be good candidates for such activities in terrestrial and aquatic habitats [9,31]. All halophytes among the flora of Qatar listed in Table 2 proved efficient in remediating various types of contaminants, including heavy metals found normally in IWW. 

However, many of these heavy metals, such as B, Co, Cu, Fe, Mn, Ni, Se, and Zn, are considered essential elements for plant nutrition [131], while other trace metals, such as Al, Cd, Cr, Hg, Pb, Sr, and others, are non-essential and toxic when their concentrations exceed certain limits [9,91,92]. These reports and articles discussed the mechanisms and roles played by these plants in phytoremediation of saline soils.

The following discussion is a brief guide for researchers, students, and decision-makers to suggest, sponsor, and develop plans and to establish road-maps for future projects to solve the problems of pollution from heavy metals and organic components of petroleum hydrocarbons. It is interesting to report that Hg and As are the most common heavy metals found in IWW at gas activities [132]. Some native plants among the flora of Qatar seem efficient in remediating Hg (Table 3). At least three halophytic plant species, namely *Cyperus* spp., *Juncus rigidus*, and *Polypogon monspeliensis*, are known to remove Hg from soils polluted during gas production [37,107,118,133]. If we look at *Polypogon monspeliensis*, this halophyte plant proved efficient in accumulating Hg in its different plant organs; therefore, it is a promising candidate for the phytoremediation of this toxic element at fields of gas facilities [120,133].

Molina et al. [133] investigated the accumulation of Hg in many native plants, including *Polypogon monspeliensis*, and they concluded that the uptake of Hg was found to be plant-specific. *Polypogon monspeliensis* proved efficient in accumulating Hg in all plant organs (roots and shoots). Moreover, this plant was reported to have taken up more than 110 times of Hg than the control plant species [120]. On the other hand, some other halophytes in Qatar have been shown to have remediating action against both Hg and As; *Salicornia europaea* is a good candidate to accumulate both heavy metals (found with petroleum hydrocarbons during gas production). The report of Al-Thani and Yasseen [9] has indicated that some native plants, such as dicots, including *Acacia* spp., *Amaranthus* spp., *Portulaca oleracea*, and *Ricinus communis*, and monocots, including *Arundo donax*, *Chloris gayana*, *Cynodon dactylon*, and *Typha domingensis*, were active in phytoremediation of this toxic trace metal. Other non-essential trace elements are found in the Qatari lands, and over time, and with continuous activities and production of gas and oil, they might accumulate substantially in the soil to levels that could have a greatly negative impact on various life aspects. *Arthrocnemum meridionale* is the most common halophyte among the flora of Qatar (Figure S4). This plant has shown a great ability to accumulate large quantities of Na^+^ and Cl^−^ in saline habitats [50]. However, its ability to accumulate heavy metals has been interesting. For example, Redondo-Gómez et al. [96] found that *Arthrocnemum macrostachyum* is a Cd-hyperaccumulator and may be useful for restoring Cd-contaminated sites, and thus it may play a significant role in the phytoremediation of soil contaminated with this metal. Moreover, it seems that such a plant might not have a barrier against the transport of this element from root to shoot (Figure 1). Accumulation of Cd negatively impact many physiological and biochemical parameters. These include impacts on growth, photosynthetic apparatus, in terms of chlorophyll fluorescence parameters, gas exchange, and photosynthetic pigment concentrations. Halophytic plants seem to have an antioxidant system and enzymatic antioxidants, which help protect them against the oxidative stress caused by high concentrations of heavy metals. For example, *Cleome gynandra* efficiently absorbs Cd and Cu; thus, it is highly recommended for phytoremediation, and should be monitored regularly during any future phytoremediation program [9]. Another example is *Halodule uninervis*, a perennial marine seagrass, that feeds marine organisms in Qatar, and was affected by oil accidentally spilled during the wars [111]. Bu-Olayan and Thomas [112] have concluded that trace metals can accumulate in the plant tissues (roots and leaves), ending up in the food chain and causing contamination of the ecosystem. *Salicornia europaea*, on the other hand, has been shown to have a great ability to accumulate heavy metals, such as Cd and Pb (found with petroleum hydrocarbons during oil production). Using such plants as fodder, or in a human diet, should be done with caution [121]. This plant uses two mechanisms of phytoremediation: the phytoextraction mechanism for Pb and Zn in the shoot system and the phytostabilization mechanism for Cu, Ni, and Cd in the root system [122].

Another halophytic plant among the flora of Qatar, *Salsola* spp. (including *S*. *soda*), showed promising potential to remediate saline soils containing various types of heavy metals, such as B, Cd, Co, Cr, Ni, Pb, Se, and Zn [113,124]. These authors suggested that after harvesting, these plants can be disposed of; however, such a solution might not be ideal as the harvested materials can cause a threat to the ecosystem if they enter the food chain. Instead, they can be incorporated into many industrial activities and recycling programs [9]. The study of Centofanti and Bañuelos [124]. evaluated the possibility of using *Salsola soda* as an alternative crop for saline soils rich with Se and B.

Many *Atriplex* spp. plants are found among the flora of Qatar, and these species and varieties are annual or short-lived perennial herbs, sub-shrubs, or shrubs. Most of these plants are fodder for camels and are common in saline soils, such as Sabkhas and salt patches at the coastline. They have adopted avoidance mechanisms through extrusion methods by developing specialized structures called salt bladders. The bladder cell of the salt bladder contains very high concentrations of salts, and eventually, the bladder cells rupture and die (see Figure 3 and Figure S1B). Such a method and concept of storing salts in bladders can be utilized and implemented in a large scheme of phytoremediation projects to clean up contaminated soils containing high salinity and heavy metals. Metal accumulation by *Atriplex* differs, based on their species and varieties and the different tissues involved, and even on the different levels of metals in the polluted soil. Moreover, these species have adopted an exclusion mechanism in the root system (see Figure 1, location B) which lets the plant retain a significant number of metals in the root tissues with little exported to the shoot system [134]. Thus, this plant, with such an ability, could be suitable to remediate highly saline soils, and could also be utilized for phytoremediation of heavy metals. This topic needs further investigation to look at the accumulation of Cd and Pb; both metals found among the IWW of oil and gas production.

*Avicennia marina* adopts different mechanisms to absorb, translocate, and accumulate heavy metals. For example, MacFarlane et al. [135] showed that Cu and Pb were accumulated actively in the root tissues, as the concentrations of these metals in the root tissues were higher than those in the sediments, while Zn accumulation was almost the same as in the sediment. On the other hand, the translocation of these elements to the top of the plant showed further differences. Cu content in the leaf tissue followed a linear relationship from lower concentrations in the sediments to higher concentrations at the top of the plant (leaves), bearing in mind that the exclusion mechanism is active to expel such elements from plants at higher sediment concentrations. Pb, on the contrary, showed little mobility toward the top of the plant, and the two main reasons behind that are (1) Pb is a non-mobile element and (2) it is excluded at different locations along the plant body, mainly between the shoot system and root system (Figure 1, location B). Zn was accumulated in the leaves at levels comparable to the concentration in the sediment. Thus, *Avicennia marina* might be suitable for the phytoremediation of a non-essential element, namely Pb.

Looking at *Cyperus* spp. they have various important uses, such as medicinal plants fodder, and range sledge, and their oils can be used as food or feed [13,45]. Phytoremediation of heavy metals has been very interesting; for example, Abdul Latiff et al. [104] found that the absorption of heavy metals by *Cyperus Kyllingia-Rasiga* was in the order of Mn > Cu > Ni > Cr > Pb > Zn > Fe > Al > Cd at a medium pH of 6.87 ± 0.71 and an electrical conductivity (EC) of 2.72 ± 1.85 µS/cm. Badr et al. [105] concluded that this plant species is a good candidate for phytoremediation of saline soils and was efficient in taking up and translocating more heavy metals (such as Cd, Cu, Ni, Co, Pb, and Zn) from roots to shoots. The high ratio of shoot to root might be the main reason behind such ability to accumulate Na^+^, Cl^−^ [3], and heavy metals, bearing in mind that an active monitoring system should be used [9].

*Halocnemum strobilaceum* is another medicinal halophyte plant that plays an important role in remediating Zn in saline soils. This plant would be appropriate in Qatari lands because pollution with excessive concentrations of Zn is possible from IWW of oil and gas activities [57,90,91]. The ability of these plants to grow in Zn-polluted and saline soils would allow them to serve the pharmaceutical industry as medicinal raw materials while playing an important role in ecological phytoremediation.

Another halophyte plant that proved efficient in remediating Zn, *Polypogon monspeliensis*, can be used to alleviate Zn toxicity in saline soils. This plant actually has double interactive effects; the study of Ouni et al. [65] concluded that many variables of growth and photosynthesis were severely reduced by this metal. However, the high concentration of salt (150 mM NaCl) alleviated the negative impact of Zn. On the other hand, Zn prevented the uptake and accumulation of Na^+^ and Cl^−^ by increasing the membrane integrity of the root surface (Figure 1, location A). A recent study by Samreen et al. [119] showed that heavy metals, such as Cr and Ni, might have a beneficial impact on many physiological and biochemical variables at certain levels. However, increasing pollution with such metals could have deleterious effects on protein content and increase proline content, as the latter response has been considered a clear sign of the stress effect. *Suaeda glauca*, another example, can tolerate heavy metals, such as Cd, Pb, and Mn, elements found in high concentrations of oil and gas activities, and physical and chemical properties of soil were significantly improved after phytoremediation [125]. Therefore, this plant species has a great ability to phyto-remediate contaminated soil containing heavy metals. The results of Al-Taisan [126] demonstrated that *Tamarix* spp. can play a significant role as vegetation (Figure S5), and for cleaning the soils of heavy metal contamination through phytoextraction. There is a desperate need to use the advantages of these plants in the phytoremediation of the environment. At the same time, continuous harvesting of their shoots could be a suitable way to recycle heavy metals [126]. Betancur-Galvis et al. [127] found that *Tamarix* spp. can resist petroleum hydrocarbons, such as polycyclic aromatic hydrocarbons (PAHs), benzo(a) pyrene, phenanthrene, and anthracene in contaminated saline soils. The growth of this plant was not affected by PAHs. With the presence of this plant in contaminated soil, the leaching of these compounds to the 32-34 cm layer decreased two-fold compared to uncultivated soil. Suska-Malawska et al. [128] confirmed that *Tamarix* spp. was efficient in the remediation of heavy metals, including Cu, Zn, Cd, and Pb. 

*Tatraena qatarensis* was recognized as a good candidate to remediate many heavy metals, such as Cd, Cr, Cu, Fe, Ni, and Zn. For example, Yaman [130] found that *Teucrium polium* proved to be a hyperaccumulator candidate for Ni, adopting a phytoextraction mechanism to extract this metal from contaminated soils. Moreover, the results of Usman et al. [129] showed that *T*. *qatarensis* is tolerant to many heavy metals, such as Cd, Cr, Cu, and Ni, thereby phyto-stabilizing them. Furthermore, Bibi et al. [136] showed that this plant represents an important source of potentially active bacteria producing antifungal metabolites of medical significance.

These heavy metals inhibit the growth and development of most glycophytes. Many aspects of physiology and biochemistry were reported to be adversely affected as follows: (1) formation of malondialdehyde (MAD), (2) overproduction of reactive oxygen species (ROS), (3) reduction of photosynthesis rate, (4) nutrition imbalance, (5) consequences of osmotic adjustment and osmoregulation, and (6) ability to regulate phytochelatins and metallothionein [137]. However, halophytes have developed structural modifications, including leaf succulence, salt glands, salt bladders, and trichomes, to alleviate ionic stresses, as shown in Figure 2, Figure 3, Figures S1, S4 and S5. These structures have different methods to avoid salt and heavy metal stresses. As an example, toxic ions are excreted through salt glands or trichomes [138], and these structures transport ions from mesophyll cells of a leaf to its surface. These ions then form crystals that are subsequently removed in various ways, such as by rain and wind [137]. Salt bladders, on the other hand, accumulate toxic ions and heavy metals, and after reaching a certain size, they burst and release their contents. The salts excreted by these methods were estimated to be 50% of the total absorbed salt [139]. Thus, extrusion and inclusion mechanisms offer many methods to keep substantial amounts of toxic salts, including heavy metals, away from these plants, or at least protect the active metabolic sites of the plant tissues from the detrimental effects of these toxic ions [140,141,142]. Recent studies suggested that salt glands and bladders might have specialized transporters to extrude heavy metals from leaf mesophyll tissues to the cavities of these structures [35]. Studies have also indicated that some microorganisms are found in these structures, which might play roles in the salt regulation of these halophytes [30,143].

### 4.2. Petroleum Hydrocarbons

Regarding the phytoremediation of organic components, including petroleum hydrocarbons, out of the twenty-six halophytic plants, only twelve plants proved efficient in remediating petroleum hydrocarbons. These plants are *Aeluropus* spp., *Avicennia marina*, *Cressa cretica*, *Cyperus* spp., *Frankenia pulverulenta*, *Halodule uninervis*, *Halopeplis perfoliata*, *Juncus rigidus*, *Polypogon monspeliensis*, *Sporobolus* spp., *Tamarix* spp., and *Teucrium polium*. A study from Iran showed that some of these halophytes proved efficient in metabolizing petroleum hydrocarbons, such as TOG (total oil and grease), near oil refinery facilities [117,118]. These pathways explained the Green Liver Model that operates in native plants [9,30] and the included references, and we assume that a similar system is functioning in halophytes in Qatar. It would be imagined that these plants can degrade the accumulated petroleum hydrocarbons that lead to useful metabolites. Therefore, research centres at universities should consider all these possibilities in any future plans to restore infected habitats.

## 5. Endophytic Microorganisms

Halophytes possess multiple mechanisms to resist salinity. These mechanisms operate as a result of genetic expressions of the inherited genetic factors (genes) that confer traits of salt resistance to these plants. However, these mechanisms are more enhanced through the action of microorganisms (bacteria and fungi) adjacent to, and associated with, these plants. These microorganisms live either in the rhizosphere or endosphere and make these native plants more resistant to salinity and possibly to other environmental factors [54]. It is the main objective of this article to look for characteristics that relate to microorganisms in the endosphere. Endophytes have been recognized as microorganisms, more specifically bacteria or fungi, that colonize the internal tissue of plants by a symbiotic or mutualistic relationship [144,145]. These microbes are found normally in roots, stems, leaves, and even in the reproductive parts, such as seeds, and these microorganisms are known not to cause any prominent negative effects on the plant’s life [146,147]. On the contrary, endophytes thrive inside the plant body to improve various functions, such as growth, physiology, and biochemistry, under extreme environmental and biotic factors. Endophytic microbes find their ways into the internal parts of the plant by two main routes: (1) vertical transmission, i.e., from generation to generation via seeds and perhaps through other plant parts, and (2) horizontal transmission, i.e., transfer from the environment to the internal plant body. These routes have been discussed in detail recently in [143,148]. These endophytes have proved to have plant growth-promoting (PGP) properties. These include multiple mechanisms: (1) direct mechanisms: nitrogen fixation, mineral (P and Fe) solubilization, siderophore production, phytohormone production (e.g., auxins, cytokinin’s, gibberellins, and ethylene), and production of stress alleviating compounds (e.g., 1-Aminocyclopropane-1-Carboxylate Deaminase), and (2) indirect mechanisms: biocontrol activities of PGPB in responding to the biotic stress by producing antibiotics [149,150]. Various types of bacteria and fungi isolates associated with many halophytic plants interact in a manner that influences many aspects of plant metabolism, physiology, and biochemistry. These include fixation of atmospheric nitrogen, solubilizing of soil nutrients, and synthesis of some natural products that protect host plants against many biotic and abiotic factors that might boost agriculture, economy, and other life aspects [9,30,143,151,152]. Therefore, studying the taxonomy, phylogeny, and activities of soil microorganisms will provide a good approach to select novel candidates that can be recognized as biological agents to improve agriculture and support the industry [153]. The following discussion explores the native halophytic plants in Qatar (Table 4) that have been shown to have endophytes, which can be utilized in phytoremediation projects for saline soils polluted with petroleum hydrocarbons and heavy metals. The roles of the associated microorganisms will be discussed, as they help remove and metabolize contaminants at the rhizosphere and endosphere. Therefore, scientists, research students, and decision-makers should be aware of the threats caused by pollution from salinity, heavy metals, and petroleum hydrocarbons. 

In Qatar, little has been done about the role of endophytes in wild plants and crops. However, it would be very useful to report that Al-Thani and Yasseen [154] have found significant counts of halo-thermophilic bacteria and cyanobacteria adjacent to the halophytic plants *Suaeda virmiculata*, *Limonium axillare*, and *Tetraena qatarensis*. However, the highest bacterial populations were found adjacent to *L*. *axillare*, followed by *T*. *qatarensis* and *S*. *virmiculata*. The bacterial cells of isolated strains were Gram-positive rods, and most of them were *Bacillus thuringiensis* or *Bacillus cereus*. These microorganisms might play a support role in alleviating salt stress and possibly other extreme environmental conditions. Such microorganisms might become part of the endosphere [148] and support plant growth and development by offering many methods and mechanisms [30] Moreover, a study by Al-Fayyad [155] found that the most common bacteria in mangrove forests (*Avicennia marina*) were Gram-positive and Gram-negative bacilli. This investigation discussed their properties and features in terms of surviving harsh environments of temperature and salinity, as well as their biochemical characterization.

From international reports, we can review the possible roles of microorganisms, bacteria, and fungi found in the most common halophytes of the flora of Qatar. It is very useful to utilize the outcomes of these reports to encourage students and researchers to conduct comprehensive investigations at the Qatari habitats to improve the ecosystem and restore the lands infected by various types of contaminants. Thus, from Table 4, the following halophyte plants might be promising candidates that serve as good examples of cooperation between endophytes and plants to mitigate the ionic stress in saline habitats, and thereby can be invested in for agricultural land and future planning.

**Table 4:** Possible endophytic bacteria associated with halophyte plants playing various roles in the flora of Qatar.

Plants	Endophytes	Roles & Characterizations	References
*Aerluropus* spp. (Monocot)	No reports	No reports	No reports
*Anabasis*. spp. (*Anabasis setifera*), (Dicot)	*Amycolatopsis anabasis*; *Aurantimonas endophytica*, *Glycomyces anabasis*	Isolated from roots	[156]
*Arthrocnemum meridionale* (Dicot)	*Bradyrhizobium* sp., *Chromohalobacter canadensis*, *Halomonas* sp., *Psychrobacter* sp., *Rudaea cellulosilytica*, Bacilli species	Bacterial consortia: isolated from different parts of the plant, many functions	[97,149,157]
*Atriplex leucoclada* (Dicot)	Various phyla, halotolerant bacteria: *Bacillus*, *Halobacillus*, and *Kocuria*	Nitrogen fixation	[158]
*Avicennia marina* (Dicot)	Large number of microbes: bacteria and fungi	Nitrogen fixation, phosphate solubilization, growth promotion in saline conditions, produces useful biological molecules	[159,160,161,162]
*Cleome* spp. (Dicot)	*Enterobacter cloacae*, *Klebsiella pneumoniae*, *Kluyvera cryocrescens*	Improves growth, establishes sustainable crop production	[163]
*Cressa cretica* (Dicot)	Bacteria and fungi, *Planctomyces*, *Halomonas*, *Jeotgalibacillus*	Rhizosphere and non-rhizosphere sources, Salt tolerant, mitigating saline stress	[54]
*Cyperus* spp. (Monocot)	Endophytic bacteria mercury resistant	Resistance to Hg, accumulate mercury	[107]
*Frankenia pulverulenta* (Dicot)	No reports	No reports	No reports
*Halocnemum strobilaceum* (Dcot)	Bacteria phyla: Actinobacteria and Firmicutes	Potential enzyme producers	[136]
*Halodule uninervis* (Monocot)	Bacteria such as: *Bacillus*, *Jeotgalicoccus*, *Planococcus*, *Staphylococcus*	Bacteria against pathogenic fungi: *Phytophthora capsici*, *Pyricularia oryzae Pythium ultimum*, *Rhizoctonia solani*	[164]
*Halopeplis perfoliata* (Dicot)	Some bacteria found in the soil associated with this species	Plays roles to improve Agriculture and industrial practices	[153]
*Halopyrum mucronatum* (Monocot)	Possible, needs investigation	No reports	No references
*Haloxylon* sp. (Dicot)	Bacteria: *Streptomyces* spp. and *Inquilinus* sp., fungi: *Penicillium* spp. are found at rhizosphere	Some other microbes thrive during phytoremediation of oil-contaminated soil	[165]
*Heliotropium* spp. (Dicot)	Endophytic fungi of various genera	Pharmaceutically significant, Natural products	[166]
*Juncus rigidus* (Monocot)	The family Sphingomonadaceae is the most abundant in the root endophytic community, other microorganisms involved	Phytoremediation: Petroleum compounds, heavy metal	[31,167]
*Limonium axillare*, spp. (Dicot)	Endophytic fungi: *Alternaria* and *Fusarium*	Might be a source of growth-promoting regulators (e.g., Gibberellines)	[168]
*Polypogon monspeliensis* (Monocot)	Rhizosphere microorganisms	Many physiological and biochemical parameters are activated, growth, and nutrition	[116,117]
*Salicornia europaea* (Dicot)	Endophytes such as *Bacillus* spp., *Planococcus rifietoensis*, *Variovorax paradoxus*, *Arthrobacter agilis*	Assistance to cope with salinity, producing 1-aminocyclopropane-1-carboxylate deaminase, Indole-3-acetic acid, Phosphate-solubilizing activities	[169,170]
*Salsola* sp. (Dicot)	Endophytes and rhizosphytes, bacteria: *Actinobacteria* & and possibly others	Bioactive secondary metabolites, production of antifungal metabolites, medical significance	[171,172]
*Seidlitzia rosmarinus* (Dicot)	Endophytes: Roots: *Brevibacterium*, *Kocuria*, *Paenibacillus*, *Pseudomonas*, *Rothia*, *Staphylococcus* Shoot: *Brevibacterium*, *Halomonas*, *Planococcus Planomicrobium Pseudomonas Rothia*, *Staphylococcus*, *Stenotrophomonas*	Improves plant fitness in saline soils, salt resistance, production of IAA, ACC (1-aminocyclopropane-1-carboxylate) deaminase, etc.	[173]
*Sporobolus* spp. (Monocot)	Fungal endophytes in the root system	Necessary for plant success in harsh environment	[174]
*Suaeda* spp. (Dicot)	Dominant phyla were Actinobacteria. Proteobacteria, Firmicutes, endophytic fungi such as *Alternaria* spp. and *Phoma* spp. were found in some species	Survival and stress resistance of the plant species.	[76]
*Tamarix* spp. (Dicot)	Various bacteria and fungi species in rhizosphere and endosphere. Bacteria: novel nickel (Ni)-resistant endophytic bacteria: *Stenotrophomonas* sp. S20, *Pseudomonas* sp. P21, and *Sphingobium* sp. S42, Fungi: *Aspergillus sydowii*, *Eupenicillium crustaceum*, *Fusarium* spp., *Penicillium chrysogenum*	Possible roles against bacteria, biotechnology roles, medical and agricultural roles	[175,176]
*Tetraena* spp. (Dicot)	Endophytic and rhizosphytic bacteria	The isolation and identification of populations of endophytic and rhizosphere bacteria, having antimicrobial potential	[136]
*Teucrium polium* (Dicot)	Two bacteria bacilli species, two fungi species, *Penicillium* spp.	Plays a role in growth and health	[177]

*Arthrocnemummeridionale*: It has been hypothesized that endophytes might play a key role in the high salt tolerance of *A*. *meridionale* [178]. Most of these endophytic bacteria belong to *Bacillus* spp., which have many functions to support this halophyte plant, including activation of enzymatic activities and increasing abilities to accumulate salts (Na^+^), thereby improving sodium phytoextraction capacity during the restoration of saline lands. Endophytes seem to enhance plant growth in saline soils. Moreover, Navarro-Torre et al. [179] found that the selected bacteria from the rhizosphere and endosphere of *A*. *meridionale* could improve the capacity of this plant, and possibly others, to remediate heavy metals (such as Cd). The study of Fouda et al. [149] confirmed this conclusion; the endophytic bacteria isolated from *A*. *meridionale* were used as an inoculant to stimulate some growth parameters of crops, such as *Zea mays*, at various stages of the life cycle.

*Avicennia marina*: Endophytes associated with this plant may play many roles to enrich the ecosystem for phytoremediation of saline wetlands. These microorganisms are also efficient in offering many methods, agents, and compounds of various types to boost the growth, physiology, and biochemistry of various plants [160]. In actuality, mangrove ecosystems are known for high productivity, as this plant is a main source of wood and could be used as camel fodder. Moreover, *A*. *marina* is rich in various important constituents, such as amino acids (e.g., Glutamic acid, Aspartic acids, Leucine, proline), fatty acids, essential minerals (Co, Cu, Fe, Mg, Mn, Na, Ni, Si, and Zn), and non-essential minerals (Cr and Pb). In addition, other organic components containing nitrogen and glycinebetaine were reported in this plant [45]. Therefore, such a plant might be a good candidate for various methods of phytoremediation of waters and soils polluted by heavy metals and high salinity levels, and is worthy of observation during its action against various types of contaminants. Janarthine and Eganathan [159] isolated some endophytic bacteria species from the inner tissues of pneumatophores of mangrove plants (*A*. *marina*) along with *Bacillus* sp. and *Enterobacter* sp. strains from the endosphere as being responsible for some important activities, such as phosphate solubilization [180], nitrogen fixation, and growth promotion. Ali et al. [160] explored the roles of endophytic bacteria from *A*. *marina* in counteracting the saline conditions in tomato (*Solanum lycopersicum*) plants. Such actions were reflected in the growth, photosynthetic pigments, and the rate of photosynthesis. This study concluded that the application of bacterial endophytes from plants growing in saline conditions can boost the plant’s salt resistance and improve its growth in such harsh environmental conditions. This study showed that the application of *Bacillus pumilus* AM11, *Exiguobacterium* sp. AM25, and some chemical agents, such as methionine, counteracted the toxicity of sodium chloride by reducing the level of lipid peroxidation and regulating antioxidants and related enzymes. 

*Cleome gynandra*: The recent work of Shipoh [163] revealed important roles played by endophytes associated with halophytic host plants, such as *Cleome* spp., to promote growth and development, as well as other aspects of life of some crop plants. Isolates of bacteria from internal tissues of this halophyte plant included *Enterobacter cloacae*, *Klebsiella pneumoniae*, and *Kluyvera cryocrescens*. When these microorganisms were used as inoculants, they exhibited various abilities to improve growth and establish sustainable crop production of rapeseed (*Brassica napus* L.). Many parameters were shown to produce plant growth regulators that contribute to ammonia production, atmospheric nitrogen fixation, fluorescence production, indole acetic acid (IAA) production, phosphate solubilization, and siderophore production, which play significant roles in improving growth, and establishing a sustainable crop yield.

*Cyperus* spp.: Endophytic bacteria associated with these species boost the phytoremediation of Hg, and such plant species are good candidates to clean contaminated soil in gas industrial facilities [107]. At least three species of the genus *Cyperus* are found among the flora of Qatar, namely: *Cyperus conglomeratus*, *Cyperus laevigatus*, and *Cyperus rotundus*. These species are good candidates for phytoremediation of soils contaminated with Hg. 

*Haloxylon* sp.: Some genera of bacteria, such as *Streptomyces* spp. and *Inquilinus* sp., and other fungal species, like *Penicillium* spp., are found at the rhizosphere of wild *Haloxylon* in the desert of Kuwait [165]. More species thrive in oil-contaminated soils; these include *Agrobacterium tumefaciens*, *Gordonia lacunae*, *Gordonia terrae*, *Lysobacter* spp., *Nocardia cyriacigeorgica*, and *Rhodococcus manshaanensis*. This study concluded that *Haloxylon salicornicum* and associated microorganisms offer high ability to clean up oil polluted soils in the desert of Kuwait. 

*Juncus acutus*: This is a good candidate for phytoremediation of pollutants with the cooperation of endophytes. Members of the bacteria belonging to the family Sphingomonadaceae showed higher relative abundance within the root endophytic communities [167]. These bacteria showed significant activities during the engineering of wetlands to remove pollutants, especially heavy metals (Cd, Ni, and Zn), from soils.

*Polypogon monspeliensis*: Rhizosphere bacteria associated with this plant were found to facilitate a substantial accumulation of Se and Hg. Such results were confirmed by the study of De Souza et al. [181], who inoculated plants with such bacteria; this caused a higher accumulation of these elements as compared to those not inoculated. García-Mercado et al. [118], in their study in Mexico, found that this plant was efficient at removing Hg from polluted soils, as this metal had polluted the lands and atmosphere as well. Hg is a predominant metal found at gas facilities during extraction and production. 

*Salicornia europaea*: Some plant growth-promoting endophytic (PGPE) bacteria were isolated from various parts of this halophyte plant: surface-sterilized roots, stems, and assimilation twigs [169]. Many of these isolates were selected for their ability to produce many components affecting plant growth, such as 1-aminocyclopropane-1-carboxylate deaminase, indole-3-acetic acid, and phosphate-solubilizing activities. Five bacterial isolates were identified, such as *Arthrobacter agilis*, *Bacillus endophyticus*, *Bacillus tequilensis*, *Planococcus rifietoensis*, and *Variovorax paradoxus*. These isolates can colonize the host plant interior tissues and, for other plants, including crops, could enhance plant growth under saline stress conditions [30,149,160,163]. Another interesting study of Furtado et al. [170] investigated the endophytic bacteria and fungi associated with *Salicornia europaea*, observing distinct communities at two different sites: (1) a polluted site with anthropogenic activities and (2) a natural saline site. The communities differ in different plant organs, i.e., the root system and shoot system. However, these communities did not show any differences between seasons, and the bacterial communities seeded to influence the fungal ones. They concluded that the endophytes of halophytes may be different from those in other plants because salinity acts as an environmental filter, and they may contribute to the host’s adaptation to adverse environmental conditions to play roles in agriculture.

*Seidlitzia rosmarinus*: Based on the report of Hadi [68], this plant is a xerophytic salt-tolerant desert plant having genes responsible for resistance to salt and drought stresses. It can serve as a very useful tool in the hands of plant breeders to produce crops resistant to these stresses. It accumulates Cu and Mn at non-toxic levels and has a high level of protein (7%) and 80% digestible organic matters [182]. With these nutritional properties, it can be used as forage for livestock, especially for camels in severe dry and saline desert conditions. Other therapeutic properties of this plant should be explored for the treatment of acne. The leaves of *S*. *rosmarinus* accumulate a large amount of soda compounds that can be used in several industries, such as making soaps and detergents, pottery, ceramics, in sugar factories (e.g., for sugar crystallization), and for copper bleaching, among other applications.

*Sporobolus* spp.: Khidir et al. [174] have shown that root-associated fungi (RAF) with many halophytes are necessary for plant success in harsh environments. Other reports from Qatar [92] unpublished data, showed that this plant might be a good candidate to remediate soil polluted with IWW from gas operations at Ras Laffan-Qatar.

*Suaeda glauca*: This plant can tolerate and accumulate heavy metals, such as Cd, Pb, and Mn, elements found in high concentrations at oil and gas operations. The physical and chemical properties of soil were significantly improved after phytoremediation [125]. Therefore, this plant species has a great ability for phytoremediation of contaminated soil containing heavy metals. Soil microorganisms associated with this halophyte plant might play an important role in the process of bioremediation.

*Tamarix* spp.: These species are salt-tolerant, and some of them are normally associated with arbuscular mycorrhizal fungi (AMF). The results of Bencherif et al. [183] have shown that inoculation with AMF boosts plant growth in moderately saline soil, which was associated with improvement in nutrition status, including nitrogen and phosphorus contents. Such results encourage researchers to cultivate *Tamarix* plants using such native inoculum. A recent study [176] isolated three novel Ni-resistant endophytic bacteria from the wetland plant *Tamarix chinensis*, and these bacteria included *Stenotrophomonas* sp., *Pseudomonas* sp., and *Sphingobium* sp. These isolates offer some growth-promoting traits, such as the production of indole acetic acid (IAA), siderophores, and 1-aminocyclopropane-1-carboxylate (ACC) deaminase [9,31]. Such activities provide the host plant with the potential to improve Ni phytoremediation. Moreover, some endophytes associated with *Tamarix* spp. offer antimicrobial activities that can be exploited in various sectors of agriculture, medicine, and biotechnology [175]. In Qatar, *Tamarix* plants were observed to thrive in some ponds near Doha city [173], personal observations.

*Teucrium polium*: This halophyte plant is associated with some endophytic bacteria and fungi to assist its growth and boost its health. Hassan [177] has reported many bacterial and fungal endophytes that have shown plant growth-promoting (PGP) properties. These included some bacteria species, such as *Bacillus cereus*, *Bacillus subtilis*, and other fungi species, such as *Penicillium chrysogenum* and *Penicillium crustosum*. These endophytes produced IAA and ammonia, showed some enzymatic and antimicrobial activities, and exhibited phosphate solubilization.

On the other hand, there are other activities these endophytes can play which should be reported here, for example:

Degradation of petroleum hydrocarbons: Farzamisepehr and Nourozi [117] found that *Polypogon monspoliensis* efficiently metabolized petroleum hydrocarbons; rhizosphere microorganisms could have a role in improving plant growth under polluted treatment. The degradation of petroleum hydrocarbons using native plants has been investigated seriously in many articles [9].

Production of metabolites: *Salsola* spp. have been proven to have immense potential for yielding useful metabolites. Bibi et al. [171] studied the endophytic and rhizospheric bacterial communities in *Salsola imbricata* for the possibility of producing bioactive secondary metabolites. Using modern technology (molecular techniques, 16S rDNA), the isolated bacterial microorganisms were grouped into four major classes: Actinobacteria, Firmicutes, β-Proteobacteria, and γ-Proteobacteria. However, the production of fungal cell wall lytic enzymes was detected mostly in members *Actinobacteria* and Firmicutes. Moreover, four bacterial strains of *Actinobacteria* with potential antagonistic activity, including two rhizobacteria, EA52 (*Nocardiopsis* sp.) and EA58 (*Pseudonocardia* sp.), and two endophytic bacteria, *Streptomyces* sp. (EA65) and *Stretomyces* sp. (EA67), were selected for secondary metabolite analyses using liquid chromatography-mass spectrometry (LC-MS). These metabolites included antibiotics such as Sulfamethoxypyridazine, Sulfamerazine, and Dimetridazole. They have concluded that this study provided an insight into antagonistic bacterial populations, especially those of *Actinobacteria* from *S*. *imbricata*, to produce antifungal metabolites of medical significance and will be characterized taxonomically in the future. Moreover, the study of Razghandi et al. [172] isolated many fungal species from *Salsola incanescens* using modern techniques. These species included *Alternaria alternata*, *A*. *chlamydospora*, *Aspergillus terreus*, *Fusarium longipes*, *Macrophomina phaseolina*, *Talaromyes pinophilus*, and *Ulocladium atrum*. These fungi species cause root or stem rotting and leaf yellowing. Moreover, other fungi that proved non-pathogenic were found as well. These included *Aspergillus niger* (induced crown swelling), *Clonostachys rosea*, *Fusarium redolens*, and *Fusarium Proliferatum* that grow as endophytic fungi. Further studies are needed to look at the roles that these endophytes play in such halophyte plants regarding their resistance to salinity and possibly other harsh environmental conditions. Additionally, Khalil et al. [162] using modern genetic techniques, identified some genera and species of fungi at the rhizosphere and endosphere of *Avicennia marina*. These microorganisms included: *Aspergillus* spp., *Chaetomium* spp., *Alternaria tenuissima*, and *Curvularia lunata*. The most potent fungus extract was analyzed using gas chromatography-mass spectrometry, verifying the presence of numerous bioactive compounds. These findings confirmed that endophytic fungal strains derived from this plant thrive in harsh ecosystems and produce bioactive metabolites, which can be recommended as a novel source for drug discovery. Moreover, Mukhtar et al. [158] have indicated that halophilic and halotolerant bacteria associated with *Atriplex* spp. and *Salsola* spp. can be used for bioconversion of organic compounds, anthropogenic or industrial, to useful products under extreme environmental stresses [9]. Regarding the endophytic fungi, Khalmuratova et al. [168] studied these species associated with *Limonium tetragonum* and other halophytic species, such as *Suaeda* spp. Fungi species belonging to *Alternaria* and *Fusarium* that are associated with these halophytes could be a source of plant growth regulators, such as gibberellins, which might be behind the thriving of halophytes in their habitats.

Antimicrobial activities: Bibi et al. [136] isolated many bacteria endophytes from plant species and other halophytes (*Avicennia marina*, *Halocnemum strobilaceum*, *Tetraena qatarensis*), and these isolates showed significant action against oomycetes fungal pathogens, such as *Phytophthora capsica* and *Pythium ultimum*. Furthermore, the results of Bibi et al. [164] showed that the sea grass *Halodule uninervis* is a common halophyte plant in the flora of Qatar, and is a good source of bacteria that proved active and capable of producing antifungal metabolites against some pathogenic fungi: *Pythium ultimum*, *Phytophthora perfoliata*, *Pyricularia oryzae*, and *Rhizoctonia solani*.

Other activities these endophytes can play: Baeshen et al. [153] found that soil associated with some halophytes such as *Halopeplis perfoliata* is rich in many bacteria species belonging to the following groups: Proteobacteria, Actinobacteria, Firmicutes, Bacteroidetes, and possibly others. These groups might play roles as biological agents to improve agricultural and industrial practices. Moreover, the study of Kuralarasi et al. [166] showed that in *Heliotropium indicum*, the main fungi species, such as *Colletotrichum*, and *Aspergillus*, were found in the endosphere, other fungi species, such as *Acremonium* spp., *Altenaria alternata*, *Bipolaris tetramera*, and *Cochliobolus lunatus* (Syn. *Curvularia lunata*), were also found in the leaves, which play important roles in pharmaceutical research and industry.

## 6. Modern Approaches

Cooperation between genetic manipulations and biological solutions has emerged as a powerful strategy for the future to solve problems of salinity, drought, and pollution. Different strategies and modern technologies have been adopted to find and develop efficient plants to desalinize soils, remove heavy metals, and metabolize petroleum hydrocarbons [3,92]. Recent trends have focused on native plants, including halophytes, and associated microorganisms, regarding the phytoremediation of polluted soils. Studies during the last decade have concluded that one of the main functions of endophytes in the plant’s life is alleviating salt stress [178], However, many other functions were also reported [184]: (1) altering plant hormone status and uptake of nutrient elements, bearing in mind that salt stress causes hormone imbalance [185] and deficiency of essential elements [186]; (2) modulating the production of reactive oxygen species (ROSs) such as O_2_^−^ (superoxide), produced as a result of inhibition of photosynthetic activity [187]. All these negative impacts of salt stress can be alleviated by: (a) increasing the activity of 1-aminocyclopropane-1-carboxylic acid (ACC)-deaminase (this enzyme reduces plant inhibitors), (b) increasing phosphate solubilization, (c) increasing nitrogen fixation [188], (d) producing indole-3-acetic acid (IAA) [189], abscisic acid (ABA), siderophores, and volatiles, and (e) increasing the production of compatible solutes to ease the negative impact of the osmotic stress of salinity [190]. Other roles might be involved and need to be explored. 

Biological approaches have emerged over the last decade to solve many problems of pollution in soil and water. These approaches have been considered environmentally friendly solutions for many problems facing the ecosystem and human life in health, agriculture, and economy. The basics of such approaches have come from the following facts: (1) the cooperation between plants and associated microorganisms to solve problems of many environmental stresses has been reported and described by many authors [3,31,191], (2) many mechanisms have been adopted by microorganisms to mitigate harsh abiotic stresses facing plants in general and crops in particular; the details of these mechanisms were discussed by [30], (3) horizontal gene transfer (HGT) is possible between microorganisms and plants; this could lead to mutually beneficial activities and boost the ecosystem to deal with harsh environments [30,192], and (4) modern biotechnology could improve, develop, and create transgenic microorganisms and plants to deal with polluted and saline soils and waters [191,193,194,195,196].

Therefore, comprehensive efforts are needed to solve the problems facing humanity regarding today’s environmental stresses and climate changes [8], as follows.

(1) Salinity problems: The selection of native plants able to regulate particular toxic ions has been considered as a new trend to desalinize soils. This subject is being investigated and surely needs biotechnological efforts in the coming years. Moreover, additional serious work is needed to find and recognize the microbial species that can boost native plants to alleviate salt stress in Sabkhas and saline patches. Some evidence was presented that some Bacilli species, adjacent to, or associated with, some halophytes, are promising in increasing the ability to accumulate Na^+^ ions and improving phytoextraction capacity during the restoration of saline lands.
(2) Heavy metal pollution: Many halophytes proved efficient in resisting heavy metals by avoidance and tolerance mechanisms. Further investigations are needed to identify more native plants that are able to select particular heavy metals from polluted soils at either oil or gas fields. Regarding As and Hg metals, at least four halophytes proved efficient to accumulate As and Hg in gas fields; these are: *Cyperus* spp., *Juncus rigidus*, *Polypogon monspeliensis*, and *Salicornia europaea*. Therefore, more studies are needed to identify some microbes that might help in increasing the capacity of native plants to accumulate these heavy metals. Moreover, other halophytes with their endophytes were reported to accumulate heavy metals, and adopting modern technology might help in increasing their capacity to deal with heavy metals in polluted soils.
(3) Organic and petroleum hydrocarbon pollution: Recent studies have shown many vital roles of some bacterial endophytes in the bioremediation (detoxification) of pollutants (organic and inorganic), plant litter, and other volatile compounds. For example, Singh et al. [184] have suggested that endophytes adapt, assemble, and colonize to promote plant growth by producing plant growth-promoting enzymes, making the host plants resistant to various environmental conditions. These enzymes include hydrolases, oxidoreductases, oxygenase, and peroxidases. These enzymes proved efficient in the degradation of pollutants [197].

## 7. Conclusions

Great pressures are placed on the social life and prosperity of people around the globe because of increasing pollution, climate change, desertification, high salinity, and health problems. These issues motivate research centers and decision-makers to find solutions for all these outstanding problems. Thus, scientists and research students in Qatar should be aware of the pollution issues caused by the expansion in industrial activities that let heavy metals and petroleum hydrocarbons accumulate in agricultural lands. One contemporary and innovative approach that could promise to solve all these problems by phytoremediation of polluted soils and waters has recently emerged using halophytes and their associated endophytes. Such microorganisms (bacteria and fungi) may provide support for the ability of these plants to cope with challenges. Moreover, such biological approaches are environmentally friendly and have proven to be efficient and sustainable. Halophytes and their endophytes could be promising candidates for phytoremediation of soils and waters polluted with industrial wastewater (IWW) in the Arabian Gulf region. Adopting modern techniques and necessary measures is required so as to conduct serious steps in securing benefits from lands after ridding them of contaminants of various kinds. Finally, the biological approach and biotechnology are promising strategies to achieve these objectives. Moreover, an active monitoring system should concentrate on the recycling of plant materials used in phytoremediation.

## Figures and Tables

**Table 2:** Halophyte plants among the flora of Qatar that could be involved in phytoremediation of heavy metals and petroleum hydrocarbon compounds.

Plants	Phytoremediation		References
	Inorganic	Organic	
*Aeluropus* spp. (Monocot)	Cd, Pb	Petroleum hydrocarbons	[92,94,95]
*Anabasis setifera* (Dicot)	Mn, Cu	No reports	[93]
*Arthrocnemum meridionale* (Dicot)	Al, Cd, Cu, Fe, Mn, Zn	*	[96,97,98]
*Atriplex leucoclada* (Dicot)	Cd, Cu, Ni, Pb, Zn	*	[99]
*Avicennia marina* (Dicot)	Cd, Co, Cr, Cu, Fe, Ni, Zn	Petroleum hydrocarbons	[100,101,102]
*Cleome* spp. (Dicot)	Efficient: (Cd, Cu)	*	[103]
*Cressa cretica* (Dicot)	Some heavy metals	Possible petroleum hydrocarbons	[37]
*Cyperus* spp. (Monocot)	Al, Cd, Co, Cr, Cu, Fe, Hg, Mn, Ni, Pb, Zn, (Phyto-stabilization of Ni)	Petroleum hydrocarbons	[104,105,106,107]
*Frankenia pulverulenta* (Dicot)	Cd, Cr, Cu, Ni, Sr, Zn	Petroleum hydrocarbons	[108]
*Halocnemum strobilaceum* (Dicot)	Cd, Cu, Fe, Mn, Ni, Pb, Zn	*	[93,109,110]
*Halodule uninervis* (Monocot)	Cu, Fe, Ni, Pb	Petroleum hydrocarbons	[111,112]
*Halopeplis perfoliata* ** (Dicot)	Some heavy metals	Possible petroleum hydrocarbons	[12,14]
*Halopyrum mucronatum* (Monocot)	Some heavy metals, bioindicator for: Cr, Fe, Pb, Zn	No reports	[113]
*Haloxylon* sp. (Dicot)	Heavy metals: Cu, Fe, Mn, Zn	Possible petroleum hydrocarbons	[93]
*Heliotropium* spp. (Dicot)	Cd, Cr, Cu, Fe, Mn, Pb, Zn	*	[114]
*Juncus rigidus* (Monocot)	Cd, Cu, Fe, Hg, Mn	Denitrification & buffering methane emission. petroleum hydrocarbons	[37,63,115]
*Limonium axillare* * (Dicot)	Cd, Co, Cr, Cu, Fe, Ni, Zn	No reports	[14]
*Polypogon monspeliensis* (Monocot)	Cr, Hg, Ni, Zn	Petroleum hydrocarbons, TOG#	[116,117,118,119,120]
*Salicornia europaea* (Dicot)	Pb, Zn, Root stabilization: Cd, Cu, Ni	No reports	[121,122]
*Salsola* sp. (Dicot)	B, Cd, Co, Cr, Cu, Fe, Mn, Ni, Pb, Se, Zn	No reports	[93,123,124]
*Seidlitzia rosmarinus* (Dicot)	Some heavy metals	No reports	[14,68]
*Sporobolus* spp. (Monocot)	Some heavy metals, and toxic ions	Petroleum hydrocarbons	[70,92]
*Suaeda* spp. (Dicot)	Cd, Cu, Fe, Mn, Pb, Zn	No reports	[93,125]
*Tamarix* spp. (Dicot)	Cd, Cu, Fe, Mn, Ni, Pb, Zn	Polycyclic aromatic hydrocarbons	[126,127,128]
*Tetraena qatarensis* (Dicot)	Cd, Cr, Cu, Fe, Ni, Zn	Possible petroleum hydrocarbons	[14,31,129]
*Teucrium polium* (Dicot)	Co, Ni	Possible petroleum hydrocarbons	[130]

* Further studies needed, ** Needs confirmation, #TOG: Total Oil and Grease.

**Table 3:** List of halophyte plants in Qatar that proved efficient in phytoremediation of heavy metals.

Metal	Plant Species	
	Monocot	Dicot
Al	*Cyperus* spp.	*Arthrocnemum meridionale*
B	-	*Salsola* sp.
Cd	*Aeluropus* spp., *Cyperus* spp., *Juncus rigidus*	*Arthrocnemum meridionale*, *Atriplex leucoclada*, *Avicennia marina*, *Cleome* spp., *Frankenia pulverulenta*, *Halocnemum strobilaceum*, *Heliotropium* spp. *Limonium axillare*, *Salicornia europaea*, *Salsola* sp., *Tamarix* spp., *Tetraena qatarensis*
Co	*Cyperus* spp.	*Avicennia marina*, *Limonium axillare*, *Salsola* sp., *Teucrium polium*
Cr	*Cyperus* spp., *Halopyrum mucronatum*, *Polypogon monspeliensis*	*Avicennia marina*, *Frankenia pulverulenta*, *Heliotropium* spp., *Limonium axillare*, *Salsola* sp., *Tetraena qatarensis*
Cu	*Cyperus* spp., *Halodule uninervis*, *Juncus rigidus*	*Anabasis setifera*, *Arthrocnemum meridionale*, *Atriplex leucoclada*, *Avicennia marina*, *Cleome* spp., *Frankenia pulverulenta*, *Haloxylon* sp., *Heliotropium* spp., *Limonium axillare*, *Salicornia europaea Salsola* sp., *Suaeda* spp., *Tamarix* spp., *Tetraena qatarensis*
Fe	*Cyperus* spp., *Halodule uninervis*, *Halopyrum mucronatum*, *Juncus rigidus*	*Arthrocnemum meridionale*, *Avicennia marina*, *Halocnemum strobilaceum*, *Haloxylon* sp., *Heliotropium* spp., *Limonium axillare*, *Salsola* sp., *Suaeda* spp., *Tamarix* spp., *Tetraena qatarensis*
Hg	*Cyperus* spp., *Juncus rigidus*, *Polypogon monspeliensis*	-
Mn	*Cyperus* spp., *Juncus rigidus*	*Anabasis setifera*, *Arthrocnemum meridionale*, *Halocnemum strobilaceum*, *Haloxylon* sp., *Heliotropium* spp., *Salsola* sp., *Suaeda* spp., *Tamarix* spp.
Ni	*Cyperus* spp., *Halodule uninervis*, *Polypogon monspeliensis*	*Atriplex leucoclada*, *Avicennia marina*, *Frankenia pulverulenta*, *Halocnemum strobilaceum*, *Limonium axillare*, *Salicornia europaea*, *Salsola* sp., *Tamarix* spp., *Tetraena qatarensis*, *Teucrium polium*
Pb	*Aeluropus* spp., *Cyperus* spp., *Halodule uninervis*, *Halopyrum mucronatum*	*Atriplex leucoclada*, *Halocnemum strobilaceum*, *Heliotropium* spp., *Salicornia europaea*, *Salsola* sp., *Suaeda* spp., *Tamarix* spp.
Se	-	*Salsola* sp.
Sr	-	*Frankenia pulverulenta*
Zn	*Cyperus* spp., *Halopyrum mucronatum*, *Polypogon monspeliensis*	*Arthrocnemum meridionale*, *Atriplex leucoclada*, *Avicennia marina*, *Frankenia pulverulenta*, *Halocnemum strobilaceum*, *Haloxylon* sp., *Heliotropium* spp., *Limonium axillare*, *Salicornia europaea*, *Salsola* sp., *Suaeda* spp., *Tamarix* spp., *Tetraena qatarensis*

-   No record.

## Supplementary Materials

The following supporting information can be downloaded at: https://www.mdpi.com/article/10.3390/plants11111497/s1, Figure S1: Diagrams showing the structures of a salt gland on leaf surface of Tamarix spp. (A), and of a salt bladder on the leaf surface of Atriplex spp. (B), Figure S2: Extra chloride ions are excluded to the sheathes of Mexican wheat plants (A: Cajeme, B: Yecora) under NaCl salinity; as exclusion mechanism to avoid its accumulation inside the active metabolic tissues, Figure S3: Much of Na+ ions are retained in roots and sheaths in Cajeme cultivar (salt tolerant cultivars) (A), while Yecora cultivar (salt sensitive cultivar) failed to do so (B); as part of physiological mechanism to avoid its accumulation in organs carrying little metabolic functions [42], Figure S4: The Arthrocnemum meridionale community lives with the parasite Cistanche phelypaea, Figure S5: Tamarix plants thrive in saline soils and polluted wetlands.
