# Supplementary material for: Endophytes and Halophytes to Remediate Industrial Wastewater and Saline Soils: Perspectives from Qatar
Source: Plants (Basel). 2022 Jun 2;11(11):1497. doi: 10.3390/plants11111497 (PMC9182595; doi:10.3390/plants11111497)
Supplement: Supplementary file 1 [file plants-11-01497-s001.zip › Supplementary Figure S1.pdf]

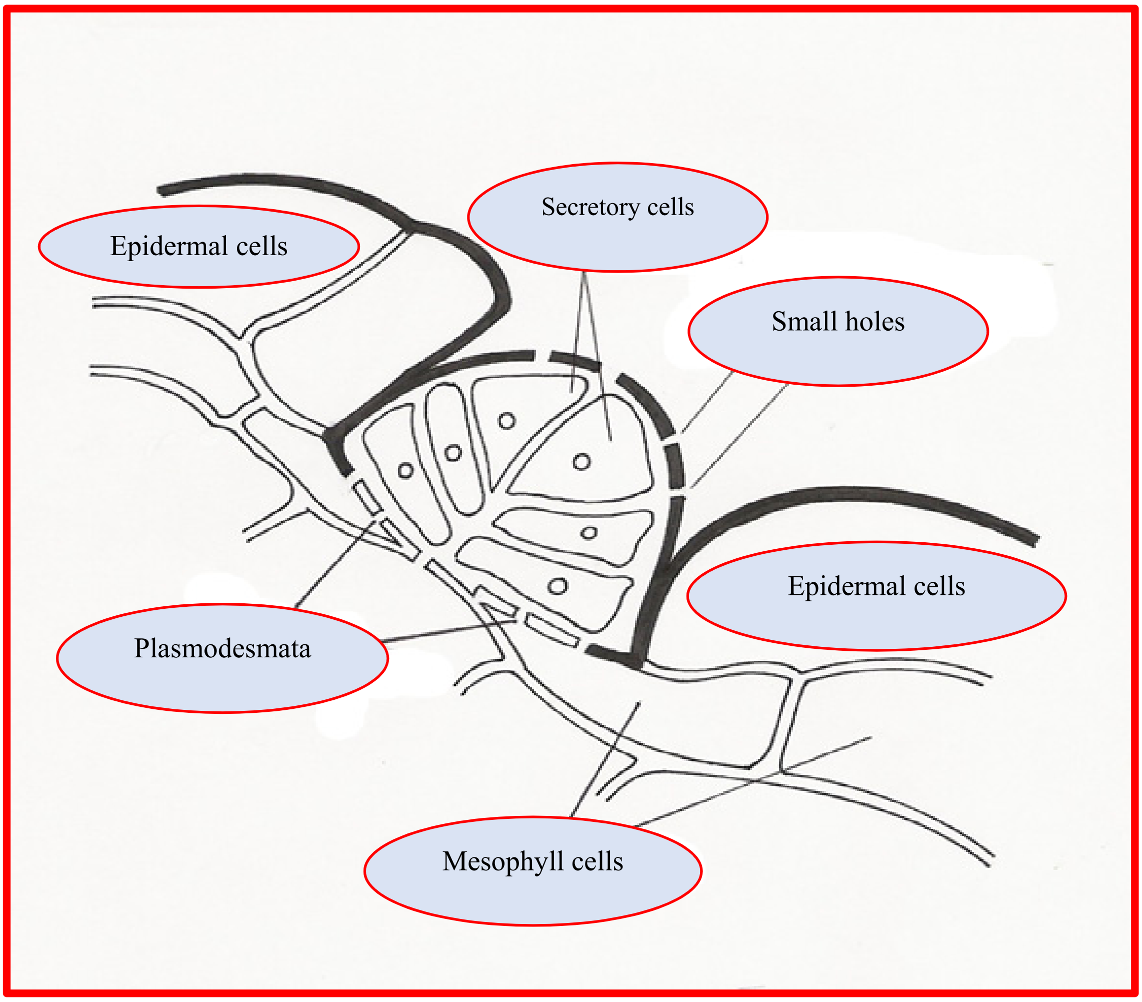
Epidermal cells
Secretory cells
Small holes
Epidermal cells
Plasmodesmata
Mesophyll cells

(A)

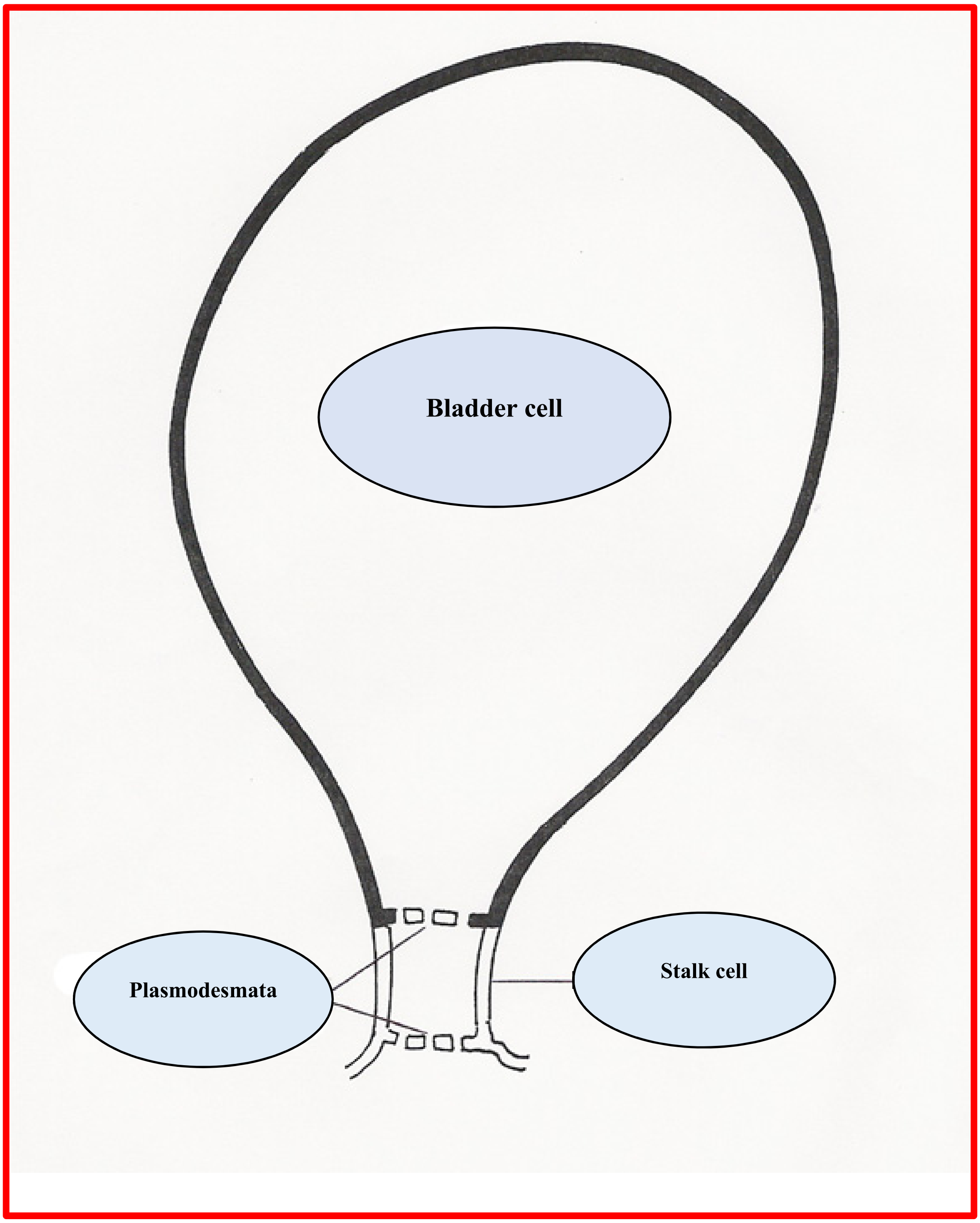

(B)

Figure S1. Diagrams showing the structures of a salt gland on leaf surface of *Tamarix* spp. (A), and of a salt bladder on the leaf surface of *Atriplex* spp. (B).
