# Supplementary material for: Endophytes and Halophytes to Remediate Industrial Wastewater and Saline Soils: Perspectives from Qatar
Source: Plants (Basel). 2022 Jun 2;11(11):1497. doi: 10.3390/plants11111497 (PMC9182595; doi:10.3390/plants11111497)
Supplement: Supplementary file 1 [file plants-11-01497-s001.zip › Supplementary Figure S2.pdf]

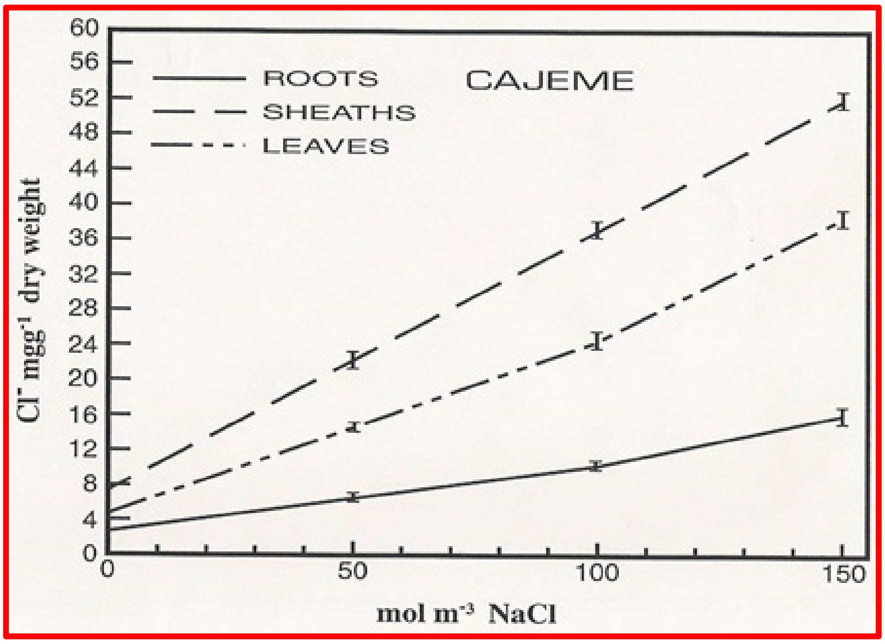

(A)

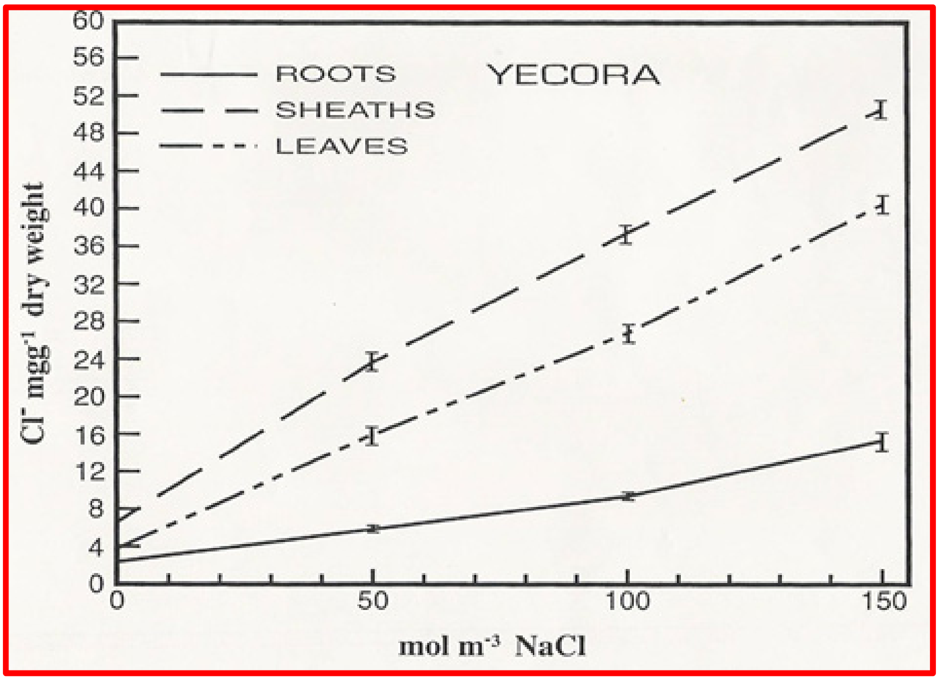

(B)

Figure S2. Extra chloride ions are excluded to the sheathes of Mexican wheat plants (A: Cajeme, B: Yecora) under NaCl salinity; as exclusion mechanism to avoid its accumulation inside the active metabolic tissues [42].
