# Supplementary material for: Endophytes and Halophytes to Remediate Industrial Wastewater and Saline Soils: Perspectives from Qatar
Source: Plants (Basel). 2022 Jun 2;11(11):1497. doi: 10.3390/plants11111497 (PMC9182595; doi:10.3390/plants11111497)
Supplement: Supplementary file 1 [file plants-11-01497-s001.zip › Supplementary Figure S3.pdf]

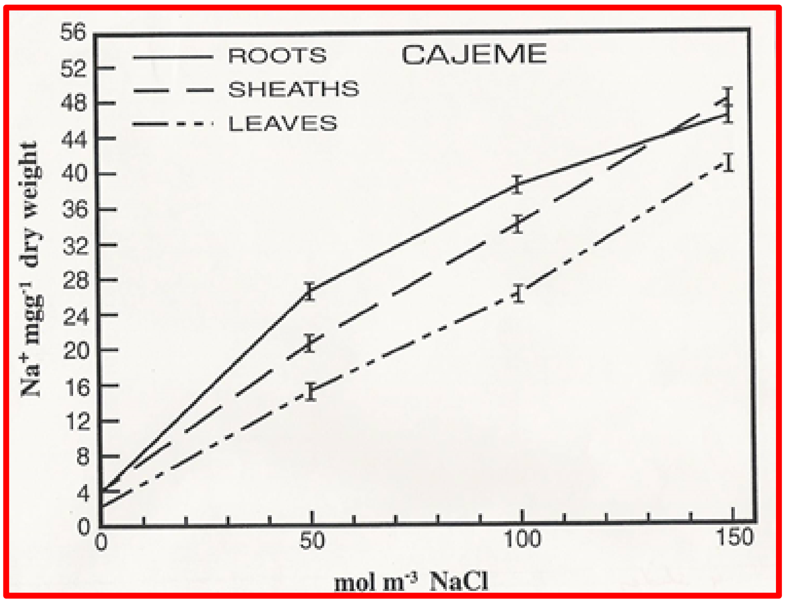

(A)

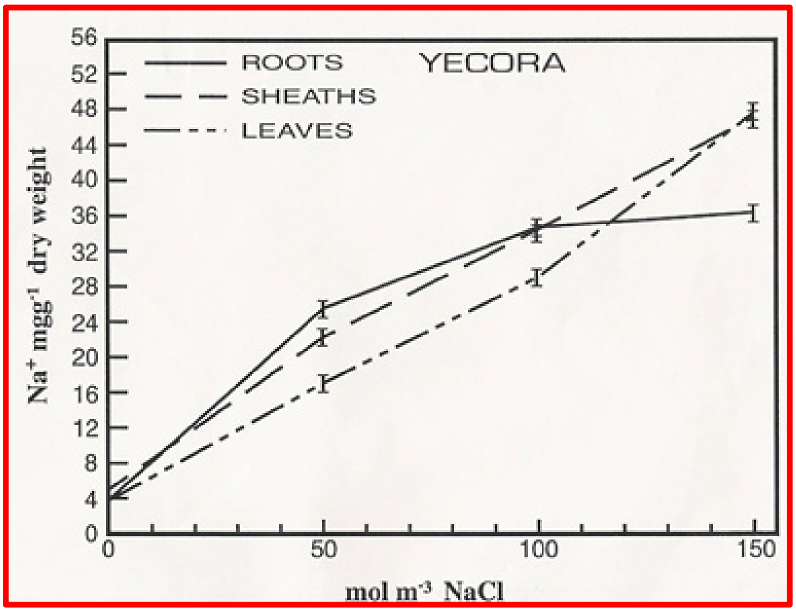

(B)

Figure S3. Much of Na^{+} ions are retained in roots and sheaths in Cajeme cultivar (salt tolerant cultivars) (A), while Yecora cultivar (salt sensitive cultivar) failed to do so (B); as part of physiological mechanism to avoid its accumulation in organs carrying little metabolic functions [42].
