# Supplementary material for: Endophytes and Halophytes to Remediate Industrial Wastewater and Saline Soils: Perspectives from Qatar
Source: Plants (Basel). 2022 Jun 2;11(11):1497. doi: 10.3390/plants11111497 (PMC9182595; doi:10.3390/plants11111497)
Supplement: Supplementary file 1 [file plants-11-01497-s001.zip › Supplementary Figure S4.pdf]

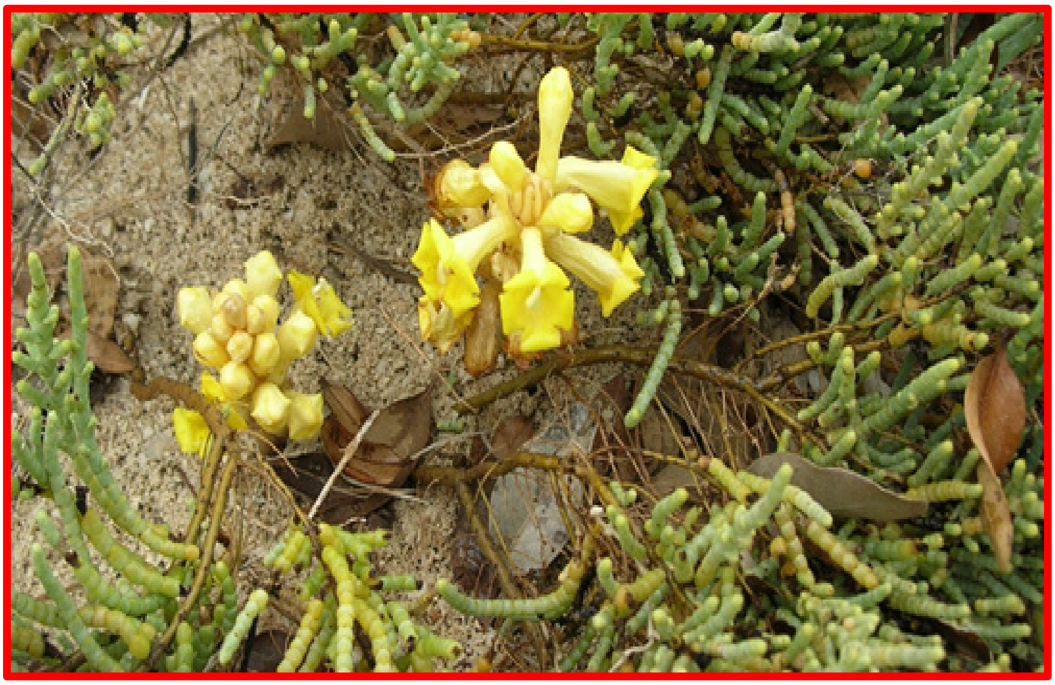

Figure S4. The *Arthrocnemum meridionale* community lives with the parasite *Cistanche phelypaea*.

N.B. *Arthrocnemum meridionale* (Ramírez & al.) Fuente & al. previously known as *Arthrocnemum macrostachyum.*
