# Supplementary figures and images for: Endophytes and Halophytes to Remediate Industrial Wastewater and Saline Soils: Perspectives from Qatar
Source: Plants (Basel). 2022 Jun 2;11(11):1497. doi: 10.3390/plants11111497 (PMC9182595; doi:10.3390/plants11111497)

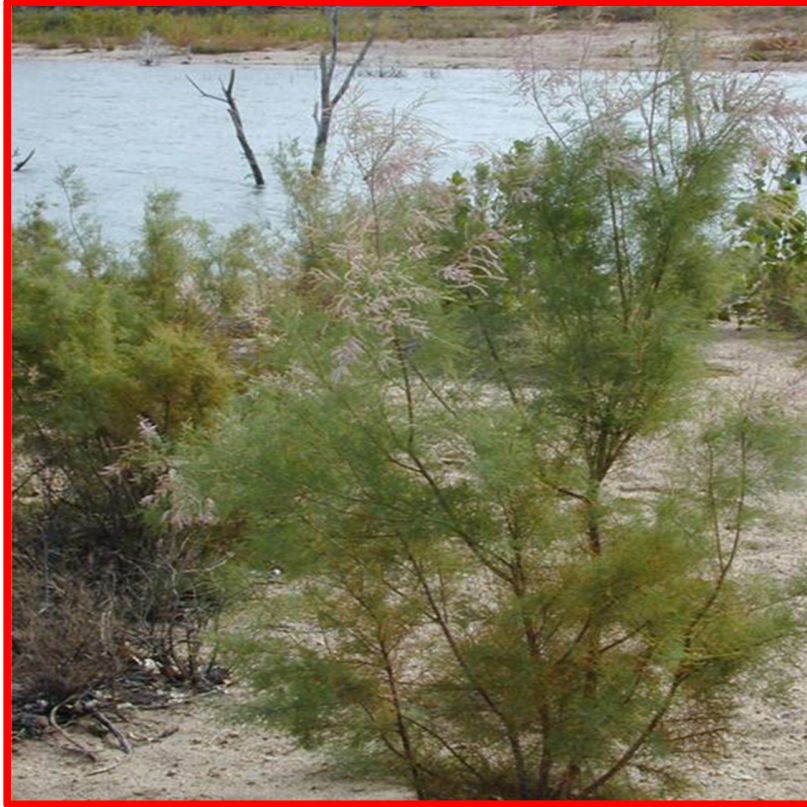

Figure S5. *Tamarix* plants thrive in saline soils and polluted wetlands.

Supplement: Supplementary file 1 [file plants-11-01497-s001.zip › Supplementary Figure S5.pdf]
